# Analyzing cell-type-specific isoform expression using IsoDiffR and long-read single-cell RNA sequencing

**DOI:** 10.1093/bioinformatics/btaf664

**Published:** 2025-12-16

**Authors:** Hu Chen, Ye Qian, Qi Dai, Ying-Feng Zheng, Zhen-Cang Zheng, Zhuo-Xing Shi

**Affiliations:** College of Life Science and Medicine, Zhejiang Sci-Tech University, Hangzhou 310018, China; State Key Laboratory of Ophthalmology, Zhongshan Ophthalmic Center, Sun Yat-sen University, Guangdong Provincial Key Laboratory of Ophthalmology and Visual Science, Guangdong Provincial Clinical Research Center for Ocular Diseases, Guangzhou 510060, China; College of Life Science and Medicine, Zhejiang Sci-Tech University, Hangzhou 310018, China; State Key Laboratory of Ophthalmology, Zhongshan Ophthalmic Center, Sun Yat-sen University, Guangdong Provincial Key Laboratory of Ophthalmology and Visual Science, Guangdong Provincial Clinical Research Center for Ocular Diseases, Guangzhou 510060, China; College of Life Science and Medicine, Zhejiang Sci-Tech University, Hangzhou 310018, China; State Key Laboratory of Ophthalmology, Zhongshan Ophthalmic Center, Sun Yat-sen University, Guangdong Provincial Key Laboratory of Ophthalmology and Visual Science, Guangdong Provincial Clinical Research Center for Ocular Diseases, Guangzhou 510060, China; Department of Critical Care Medicine, Taizhou Central Hospital (Taizhou University Hospital), Taizhou, Zhejiang 318000, China; State Key Laboratory of Ophthalmology, Zhongshan Ophthalmic Center, Sun Yat-sen University, Guangdong Provincial Key Laboratory of Ophthalmology and Visual Science, Guangdong Provincial Clinical Research Center for Ocular Diseases, Guangzhou 510060, China

## Abstract

**Motivation:**

The analysis of RNA isoforms using long-read single-cell RNA sequencing (scRNA-seq) represents a frontier in gene expression research, offering deeper insights beyond traditional gene-level analysis. However, specialized analytical methods tailored for this advanced technology remain scarce, underscoring the urgent need for novel tools to match the rapid pace of its development.

**Results:**

Here, we present IsoDiffR, a robust tool designed to identify RNA isoforms with expression patterns that differ from their corresponding genes or major isoforms across cell types, enabling both pairwise and multicell-type comparisons. Using IsoDiffR, we conducted benchmark tests using simulated data and analyzed long-read scRNA-seq data from the corneal limbus of *Macaca fascicularis* and the human frontal cortex, uncovering previously unrecognized cell-type-specific isoforms that were not detectable using conventional approaches. Additionally, we explored the structural and functional properties of these isoforms, from their nucleotide sequences to their corresponding protein isoforms, revealing their potential biological roles. Our findings offer new perspectives on gene expression regulation at the single-cell level and provide a methodological framework for future investigations of isoform-specific functions in diverse biological contexts.

**Availability and implementation:**

The R package is available on https://github.com/Eveqian98/IsoDiffR.

## 1 Introduction

In recent years, advancements in long-read scRNA-seq technology have enabled high-throughput, high-accuracy analysis of RNA isoform expression and structural characteristics across thousands of individual cells (Saliba *et al.* 2014, Zappia *et al.* 2018, Chen *et al.* 2019, Luecken and Theis 2019, Kharchenko 2021). Unlike short-read sequencing, long-read scRNA-seq technology provides precise insights into RNA isoform expression at the single-cell level, offering not only gene-level expression profiles but also detailed isoform-specific information. This capability has opened new avenues for understanding the complexity of transcriptomes in diverse cellular contexts, enhancing our ability to explore the functional diversity of RNA isoforms (Gupta *et al.* 2018, Vu *et al.* 2018, Wu *et al.* 2020, Pan *et al.* 2022).

While previous studies have focused on single-exon splicing events or percent-spliced in (PSI) metrics, which have yielded valuable insights (Hu *et al.* 2020a,b; Westoby *et al.* 2020, Hazzard *et al.* 2022), these approaches may not fully capture the functional roles of RNA isoforms. Isoforms often exert their biological functions through their complete sequence structures, necessitating analytical methods that consider full-length RNA isoforms as the primary unit of analysis. Such approaches would allow for a more comprehensive understanding of the complex regulatory mechanisms governing RNA splicing. Furthermore, RNA isoform expression matrices derived from single-cell RNA sequencing data offer the potential to uncover cell-type-specific expression patterns. However, distinguishing between gene-driven and isoform-driven expression remains a significant challenge. Developing more sophisticated methods to analyze RNA isoform expression at the single-cell level is therefore essential for advancing our understanding of the intricate mechanisms underlying gene regulation.

Moreover, similar to the analysis of marker genes in gene expression matrices (Gribov *et al.* 2010, Hu *et al.* 2023), RNA isoform expression matrices derived from scRNA-seq data can provide valuable insights into cell-type-specific isoform expression patterns. However, these analyses often face the challenge of distinguishing whether observed isoform-specific expression arises from differential gene expression across cell types or from isoform-specific regulatory mechanisms. While existing tools such as IsoformSwitchAnalyzeR (Vitting-Seerup and Sandelin 2019) and tappAS (de la Fuente *et al.* 2020) designed to analyze pairwise isoform switching and usage, their application to long-read scRNA-seq data remains limited. This underutilization restricts their ability to address more complex biological questions and fully exploit the potential of long-read sequencing technologies.

Here, we present IsoDiffR, a specialized software package developed for the analysis of single-cell RNA isoform expression data. IsoDiffR processes single-cell RNA isoform expression matrices and facilitates the identification of differentially expressed isoforms (DEIs) across both pairwise and multicell-type comparisons. By integrating adjusted R-squared (adj.*R*^2^) and cosine similarity analyses with assessments of isoform-to-gene expression ratio differences across cell types, IsoDiffR offers a robust framework for detecting isoform-level expression variations. The primary goal of IsoDiffR is to identify RNA isoforms that exhibit expression patterns distinct from their corresponding gene-level expression or major isoforms, thereby uncovering isoforms with unique expression profiles and functional significance across diverse cell types.

We conducted benchmark tests using simulated long-read bulk and single-cell isoform sequencing data, which revealed that IsoDiffR outperforms existing software. Leveraging IsoDiffR, we identified a range of RNA isoforms exhibiting distinct expression patterns across cell types in datasets derived from the corneal limbus of *Macaca fascicularis* and the human frontal cortex. Furthermore, we used AlphaFold3 (Abramson *et al.* 2024) to compare the splicing structures and predicted protein conformations of these isoforms with those of their respective major isoforms, revealing substantial structural differences. Further analyses indicated that these structural variations may influence protein-protein interactions. This approach offers novel insights and advanced methodological tools for probing cell-type-specific functional mechanisms at the isoform level.

## 2 Materials and methods

### 2.1 Datasets

The long-read scRNA-seq data from the *Macaca fascicularis* corneal limbus were sourced from the study by Shi *et al.* (2023), while the human frontal cortex data were obtained from the study by Hardwick *et al.* (2022).

### 2.2 Data processing and downstream analysis

Long-read scRNA-seq data were processed using scISA-Tools, including preprocessing, isoform identification, and generation of gene- and isoform-level count matrices (Shi *et al.* 2023). Only annotated isoform categories—FSM (fully spliced matching), ISM (incomplete spliced matching), NIC (novel in catalog), and NNC (novel not in catalog)—were retained. The resulting matrices were imported into Seurat (v4.3.0) for downstream analyses. Cells with <500 detected genes, >10% mitochondrial content, or genes expressed in <5 cells were removed. Each dataset was normalized using the LogNormalize method, and batch effects across samples were corrected using canonical correlation analysis (CCA).

For clustering, the top 2000 highly variable genes were selected for principal component analysis (PCA). The first 10 principal components from both gene- and isoform-level matrices were used to construct a shared nearest-neighbor (SNN) graph, and clusters were identified using FindClusters with a resolution of 0.15 (Hao *et al.* 2021). Marker genes and isoforms were identified using FindAllMarkers, applying |avg_logFC| > 1 and *P* < .05 as selection criteria.

### 2.3 IsoDiffR workflow

#### 2.3.1 Identification of candidate DEIs

Marker isoforms from *Macaca fascicularis* limbal cells and human frontal cortex were first identified using Seurat’s FindAllMarkers and used as candidates for DEI analysis. In IsoDiffR, isoforms were filtered using the min.pct criterion (default 0.25) and by requiring each isoform to contribute >10% of its parent gene’s expression. For improved regression model performance, IsoDiffR’s subset_ident function was used to restrict the analysis to selected cell types, typically limited to 3–5 groups.

#### 2.3.2 Identification of multi-DEI

In more than two cell types, we stored the CPM values of the isoform, its corresponding gene, and the major isoform in vectors for each cluster. The explanatory power of the isoform on the gene and major isoform was measured using the statistic adj. *R*^2^ in linear regression analysis, which reflects the goodness of fit between the isoform expression curve and the gene/major isoform expression curve. The calculation formula for adj. *R*^2^ is as follows:


$$R_{\mathrm{adj}}^{2}=1-\;\frac{\left(1-\frac{{\left(\Sigma_{i=1}^{n}(x_{i}-\bar{x})(y_{i}-\bar{y})\right)}^{2}}{{\Sigma_{i=1}^{n}(x_{i}-\bar{x})}^{2}{\Sigma_{i=1}^{n}(y_{i}-\bar{y})}^{2}})(n-1\right)}{n-k-1}$$

In this context, x and y represent the expression vectors of the gene or major isoform across various cell types and the isoform itself across the same cell types, respectively. The n represents the sample size, which is the number of selected cell types, and since there is only one predictor variable, k=1 remains constant. The closer of $R_{\mathrm{adj}}^{2}$ to 1, the better the isoform explains the gene and major isoform, indicating higher consistency in expression. In IsoDiffR, isoforms with a $R_{\mathrm{adj}}^{2}$ value of <0.2 are considered candidates for multi-DEI. Finally, we define the ratio range as the range of the isoform expression relative to the corresponding gene expression across clusters. Isoforms with a ratio range below a threshold (default value 0.05) are filtered out. By applying these two filtering methods, isoforms exhibiting differential expression between multiple clusters compared to their corresponding gene or major isoform are identified and defined as multi-DEIs.

#### 2.3.3 Identification of pair-DEI

When searching for DEIs between two cell types, we first examine the consistency of the expression trends of the isoform and its corresponding gene or major isoform across the two clusters using the Pearson Correlation Coefficient. The formula for calculating the Pearson Correlation Coefficient is as follows:


$$r_{xy}=\frac{\sum_{i=1}^{n}\left(x_{i}-\bar{x})(y_{i}-\bar{y}\right)}{\sqrt{\sum_{i=1}^{n}{\left(x_{i}-\bar{x}\right)}^{2}\sum_{i=1}^{n}{\left(y_{i}-\bar{y}\right)}^{2}}}$$

Since the vector length is 2 when comparing two cell types, the result of $r_{xy}$ can only be 1 or −1. When $r_{xy}$=1, it indicates that the expression trends of the two are consistent, whereas when $r_{xy}$=−1, it suggests that the expression trends are opposite. We then introduce Cosine Similarity to assess the similarity of the vector directions between the isoform and the major isoform or gene based on their CPM values across the two clusters. The formula for Cosine Similarity is as follows:


$$\cos\left(\theta \right)=\frac{\Sigma_{i=1}^{n}x_{i}y_{i}}{\sqrt{\Sigma_{i=1}^{n}x_{i}^{2}}\cdot \sqrt{\Sigma_{i=1}^{n}y_{i}^{2}}}$$

We multiply the Pearson Correlation Coefficient by Cosine Similarity and use the resulting value to assess the similarity of isoform expression trends with those of the major isoform or gene in two clusters. The threshold is set based on the sign of the value, with positive values selected if <0.9, and negative values selected if > −1. Both parts are then considered as candidate pair DEIs. Additionally, we introduce the ratio difference to define the difference in the proportion of isoform expression relative to the gene expression in the two clusters, applying further filtering. This process ultimately identifies isoforms that show differential expression compared to the major isoform or gene between the two clusters, and these isoforms are defined as pair-DEIs in IsoDiffR.

Additionally, when defining isoforms with differential expression relative to the major isoform, we also remove cases where the major isoform itself exhibits differential expression compared to the gene.

### 2.4 Simulation of RNA-seq datasets for benchmarking

To benchmark differential transcript detection, we constructed a simulation framework using both bulk and single-cell settings. A total of 1000 genes were simulated, each with three isoforms, one designated as differential and the others as nondifferential. Differential isoforms were defined by expression patterns across two conditions that diverged from gene-level or nondifferential isoform trends, spanning mild to strong and reversed effects.

For bulk simulations, two conditions with two biological replicates each were generated. Noisy long-read data were produced using IsoSeqSim at sequencing depths of 0.5M, 1M, and 2M reads, yielding 12 datasets. Isoform abundances were quantified with IsoQuant (Prjibelski *et al.* 2023) and used as input for downstream analyses.

Single-cell datasets were simulated from bulk profiles using a negative binomial model with expression-dependent dropout, capturing biological heterogeneity and scRNA-seq sparsity in line with principles implemented in Splatter (Zappia *et al.* 2017) and scDesign2 (Sun *et al.* 2021, Song *et al.* 2024). Cell numbers of 300, 500, and 1000 were generated at multiple depth settings, producing 36 single-cell datasets for benchmarking.

Performance was assessed using the predefined differential isoforms as ground truth. Precision, recall, and F1-scores were computed for both bulk and single-cell datasets to evaluate accuracy and robustness.

### 2.5 Empirical FDR estimation via cell-type label permutation

To evaluate the robustness of differential isoform detection, we performed empirical false discovery rate (FDR) analysis on both real and simulated datasets. In the simulated datasets, where the ground truth of differential isoforms is known, the FDR at each threshold was calculated directly as the proportion of detected isoforms that were not truly differential. For the real datasets-comprising publicly available PacBio single-cell long-read RNA-seq data from human PBMCs-we used a label permutation approach. Specifically, the cell-type label of each cell was randomly permuted 100 times at each detection threshold while preserving the original expression profiles. Isoforms identified as differentially expressed under these permuted labels were regarded as false positives. The empirical FDR at threshold *t* was then defined as the mean FDR across all permutations, given by:


$$\mathrm{FDR}(t)=\frac{\overset{-}{N_{\mathrm{FP}}(t)}}{N_{\mathrm{detected}}(t)}$$

where $\overset{-}{N_{\mathrm{FP}}(t)}$ denotes the average number of differential isoforms identified across all permutations (false positives), and $N_{\mathrm{detected}}(t)$ is the total number of isoforms detected at threshold t.

### 2.6 Functional enrichment and protein characterization analyses

Gene Ontology (GO) and KEGG pathway enrichment analyses were performed using the clusterProfiler package (v4.6.2) (Wu *et al.* 2021). with significance defined as *P* < .05. Results were visualized using ggplot2. Gene annotations for the *Macaca fascicularis* corneal limbus dataset were obtained from AnnotationHub (AH107668), whereas human frontal cortex genes were annotated using org. Hs.eg.db (AH73877).

Protein sequences corresponding to selected genes were aligned using CLUSTALW (Thompson *et al.* 2003), and structural features were visualized through ENDscript (v3.0) (Robert and Gouet 2014).

Conserved protein domains were annotated using the NCBI Conserved Domain Database (CDD). Protein structural models—either single-chain or multimeric—were predicted using AlphaFold3 (Abramson *et al.* 2024) and subsequently aligned and visualized in PyMOL (v2.5.7) (DeLano 2002).

### 2.7 Protein interaction network analysis

Protein-protein interactions were retrieved from the STRING database and imported into Cytoscape (v3.9.1) (Smoot *et al.* 2011) for network construction, visualization, and identification of putative core regulatory components.

## 3 Results

### 3.1 Overview of methodology and experimental design

**Figure 1.**
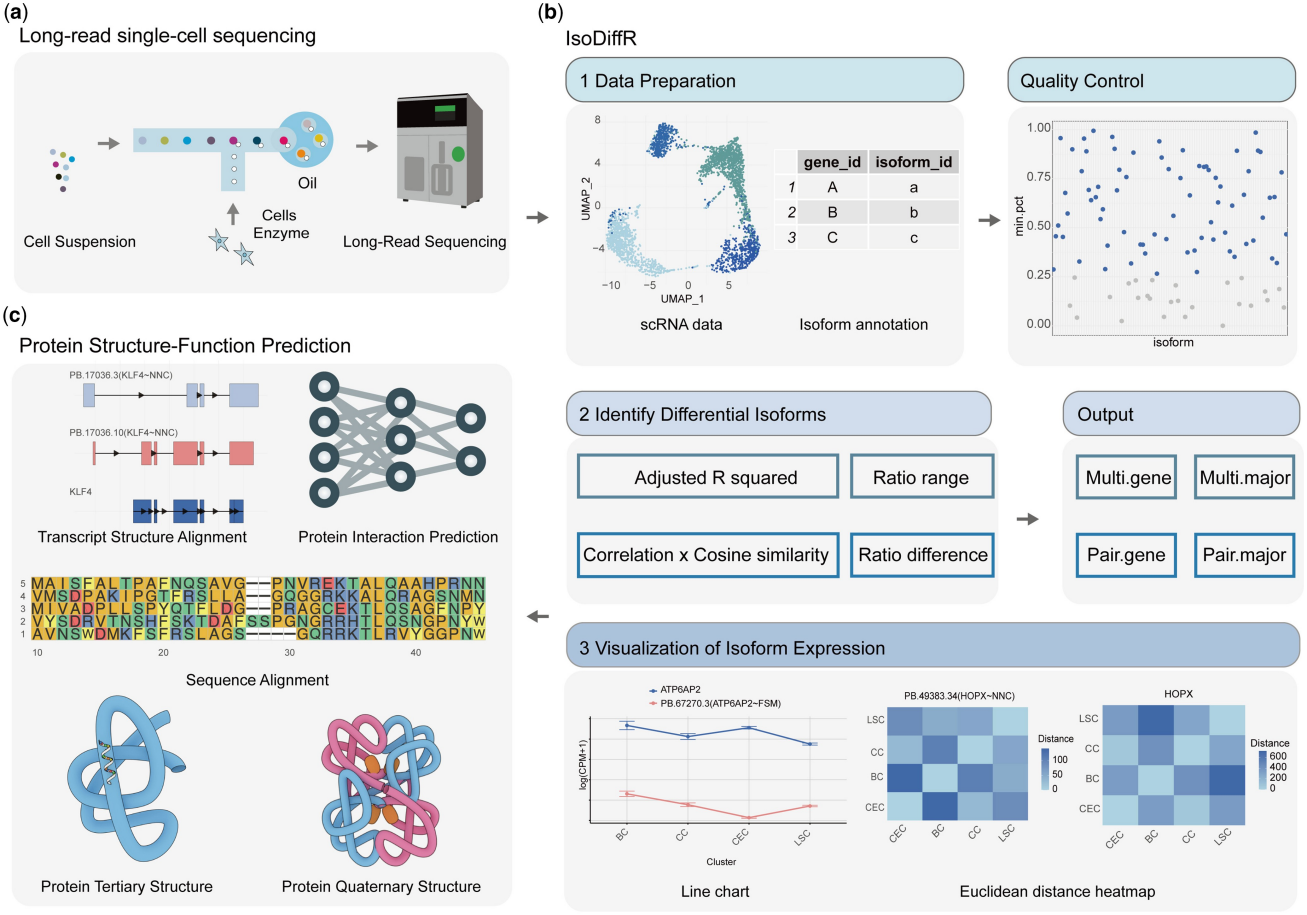
Overview of the experimental workflow. (a) Acquisition of long-read scRNA-seq data from the corneal limbus of *Macaca fascicularis*. (b) The R package IsoDiffR consists of three main parts: data preparation, identification of DEIs, and visualization of isoform expression. (c) Functional analysis and protein prediction of DEIs.

Following long-read scRNA-seq (Fig. 1a), the IsoDiffR workflow (Fig. 1b) begins with two input datasets: a single-cell RNA isoform Seurat object with cell type annotations and a GTF file containing gene and transcript IDs. Marker isoforms for each cell type are identified using Seurat’s “*FindAllMarkers*” function, with a “min.pct” threshold to exclude lowly expressed isoforms and a filter for isoforms contributing >10% of the corresponding gene’s expression. These filtered markers are considered candidate DEIs for further analysis against corresponding genes or major isoforms.

CPM values of candidate isoforms and their corresponding genes or major isoforms are extracted across cell types to form numerical vectors. The isoform CPM vector serves as the dependent variable (*x*), while the gene or major isoform CPM vectors are independent variables (*y*). DEIs are categorized as multi-DEIs (differential across multiple cell types) or pair-DEIs (differential between two cell types). For multi-DEIs, the adjusted *R*^2^ from linear regression quantifies divergence from gene or major isoform expression patterns. For pair-DEIs, the product of Pearson correlation and cosine similarity evaluates consistency or deviation between the two vectors. Additionally, the change in isoform-to-gene expression ratio is considered to filter DEIs with minimal relative variation.

IsoDiffR provides two visualization approaches: line plots highlighting differential expression trends relative to genes or major isoforms across cell types, and Euclidean distance-based heatmaps representing expression differences between isoforms, genes, or major isoforms. Default thresholds were validated through empirical FDR estimation, showing consistently low false discovery rates in both label-permuted and simulated datasets (Fig. 1, available as supplementary data at *Bioinformatics* online).

To benchmark performance, IsoDiffR was applied to simulated bulk and single-cell RNA-seq datasets and compared against ground-truth labels (Tables 1–2, available as supplementary data at *Bioinformatics* online). Pseudo-bulk data from the *Macaca fascicularis* corneal limbus were used to compare DEIs identified by IsoDiffR with switch isoforms detected by IsoformSwitchAnalyzeR. IsoDiffR was further applied to single-cell datasets from the Macaca corneal limbus and human frontal cortex. Selected DEIs underwent in-depth structural and functional characterization, including GO and KEGG pathway enrichment, as well as protein structure prediction with AlphaFold3, to explore their biological roles (Fig. 1c).

### 3.2 Benchmarking with simulated data validates the accuracy of IsoDiffR

**Figure 2.**
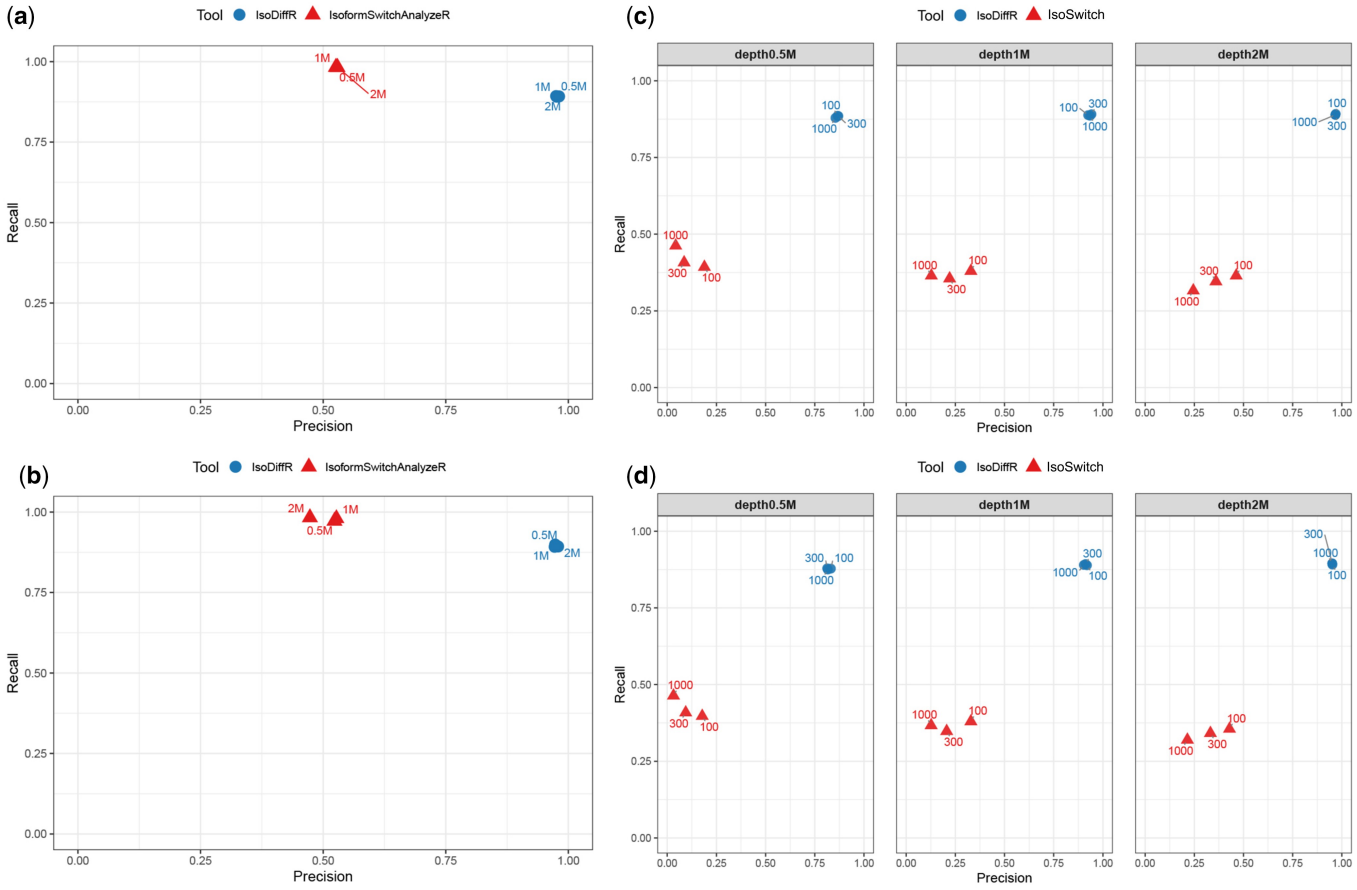
Comparison of IsoDiffR with IsoformSwitchAnalyzeR and IsoSwitch on Simulated Data. (a, b) Bulk RNA-seq simulations at three sequencing depths for two independent samples, showing IsoDiffR versus IsoformSwitchAnalyzeR. (c, d) Single-cell RNA-seq simulations across three sequencing depths and three cell counts for two independent samples, comparing IsoDiffR with IsoSwitch.

To evaluate IsoDiffR’s accuracy and robustness, we benchmarked it against IsoformSwitchAnalyzeR on simulated bulk RNA-seq datasets with sequencing depths of 0.5M, 1M, and 2M reads per sample, each with two biological replicates per condition. IsoformSwitchAnalyzeR exhibited high recall but low precision (∼0.5), indicating a substantial false-positive rate. In contrast, IsoDiffR consistently achieved high precision and recall across all sequencing depths, demonstrating reliable differential isoform detection in bulk RNA-seq (Fig. 2a and b; Table 1, available as supplementary data at *Bioinformatics* online).

We further assessed single-cell performance by comparing IsoDiffR to IsoSwitch, using simulated scRNA-seq datasets with sequencing depths of 0.5M, 1M, and 2M reads and 100, 300, or 1000 cells per group. IsoSwitch showed limited performance, with precision and recall near 0.5 under all conditions. IsoDiffR maintained high and stable precision and recall across all settings, highlighting its robustness in single-cell RNA-seq (Fig. 2c and d; Table 2, available as supplementary data at *Bioinformatics* online).

### 3.3 Evaluation of IsoDiffR and IsoformSwitchAnalyzeR on pseudo-bulk datasets

**Figure 3.**
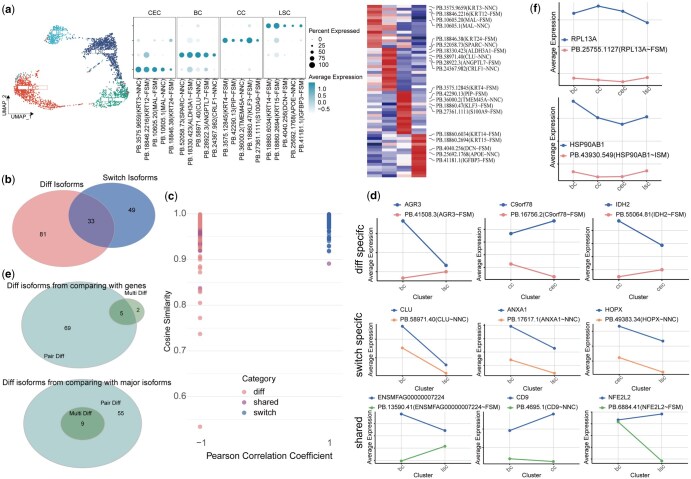
Benchmarking IsoDiffR against IsoformSwitchAnalyzeR on pseudo-bulk data from *Macaca fascicularis* corneal limbus. (a) Single-cell atlas of the long-read dataset (UMAP, dot plot, heatmap). (b) Numbers and distributions of DEIs identified by IsoDiffR in pairwise cell-type comparisons, alongside switch isoforms detected by IsoformSwitchAnalyzeR. (c) Distribution of the Pearson correlation × cosine similarity values for isoforms unique to IsoDiffR, unique to IsoformSwitchAnalyzeR, or shared. (d) Representative expression profiles of isoforms unique to IsoDiffR, unique to IsoformSwitchAnalyzeR, and shared isoforms, compared with their corresponding genes. (e) Numbers and distributions of DEIs in pairwise and multigroup comparisons, compared with genes and major isoforms. (f) Representative multigroup DEIs contrasted with their corresponding genes.

IsoformSwitchAnalyzeR is a powerful tool for detecting RNA isoform switches at the bulk data level. To compare IsoDiffR with IsoformSwitchAnalyzeR, we applied both tools to a published long-read scRNA-seq dataset from the corneal limbus of *Macaca fascicularis* (Shi *et al.* 2023). Cell clustering at gene and isoform levels identified distinct populations: corneal epithelial cells (CEC), basal cells (BC), conjunctival cells (CC), and limbal stem cells (LSC) (Fig. 3a, Fig. 2, available as supplementary data at *Bioinformatics* online). Marker genes and isoforms specific to these populations were used for differential expression analyses.

We generated pseudo-bulk datasets by averaging isoform expression within each cell type across three sampling rounds. IsoDiffR identified 114 DEIs, IsoformSwitchAnalyzeR detected 82 switch isoforms, with 26 shared between the tools (Fig. 3b, Table 3, available as supplementary data at *Bioinformatics* online). Isoforms uniquely detected by IsoformSwitchAnalyzeR exhibited near-perfect correlation and cosine similarity with their corresponding genes (*r*_Xγ_ ≈ 1, cos(θ) > 0.9), indicating limited divergence in expression patterns (Fig. 3c).

**Figure 4.**
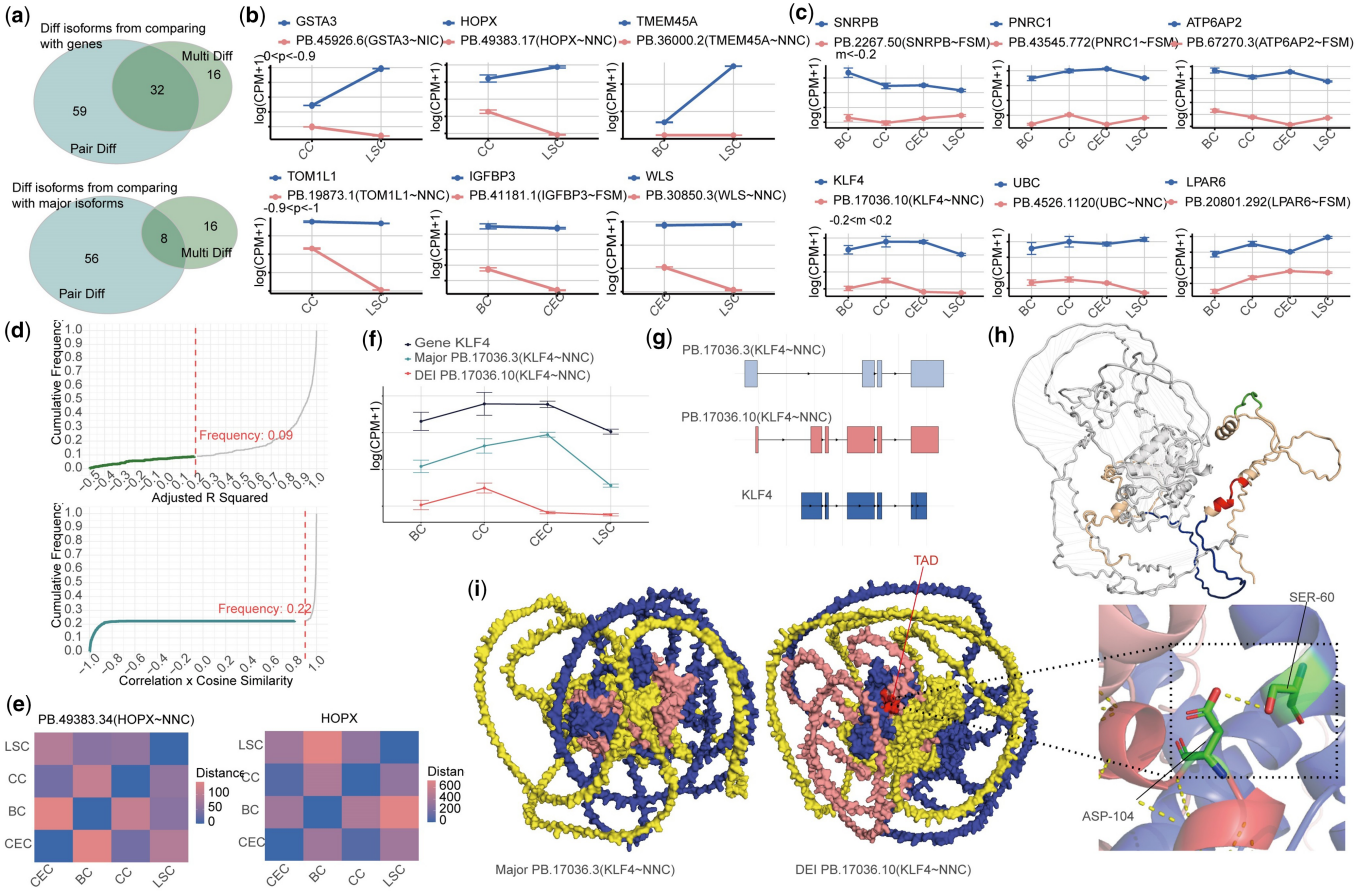
IsoDiffR analysis of *Macaca fascicularis* corneal limbus long-read scRNA-seq data. (a) Set relationships of pairwise (pair-DEIs) and multicell-type DEIs comparing isoforms with corresponding genes (top) or major isoforms (bottom). (b) Representative expression patterns of three multi-DEIs from two adj. *R*^2^ ranges across cell types. (c) Representative expression patterns of three pair-DEIs from two correlation × cosine similarity ranges. (d) Cumulative distributions of adj. *R*^2^ (left) and correlation × cosine similarity (right) for all isoforms. (e) Euclidean distance heatmaps of a representative multi-DEI (left) and its corresponding gene (right). (f) Example of a DEI with a distinct expression pattern. (g) Expression comparison of the DEI with its corresponding gene and major isoform. (h) Structural comparison of DEI and major isoform proteins, highlighting DEI-specific features. (i) Predicted interactions of DEI and major isoform proteins with transcriptional co-activators, emphasizing functional residues in the DEI.

**Figure 5.**
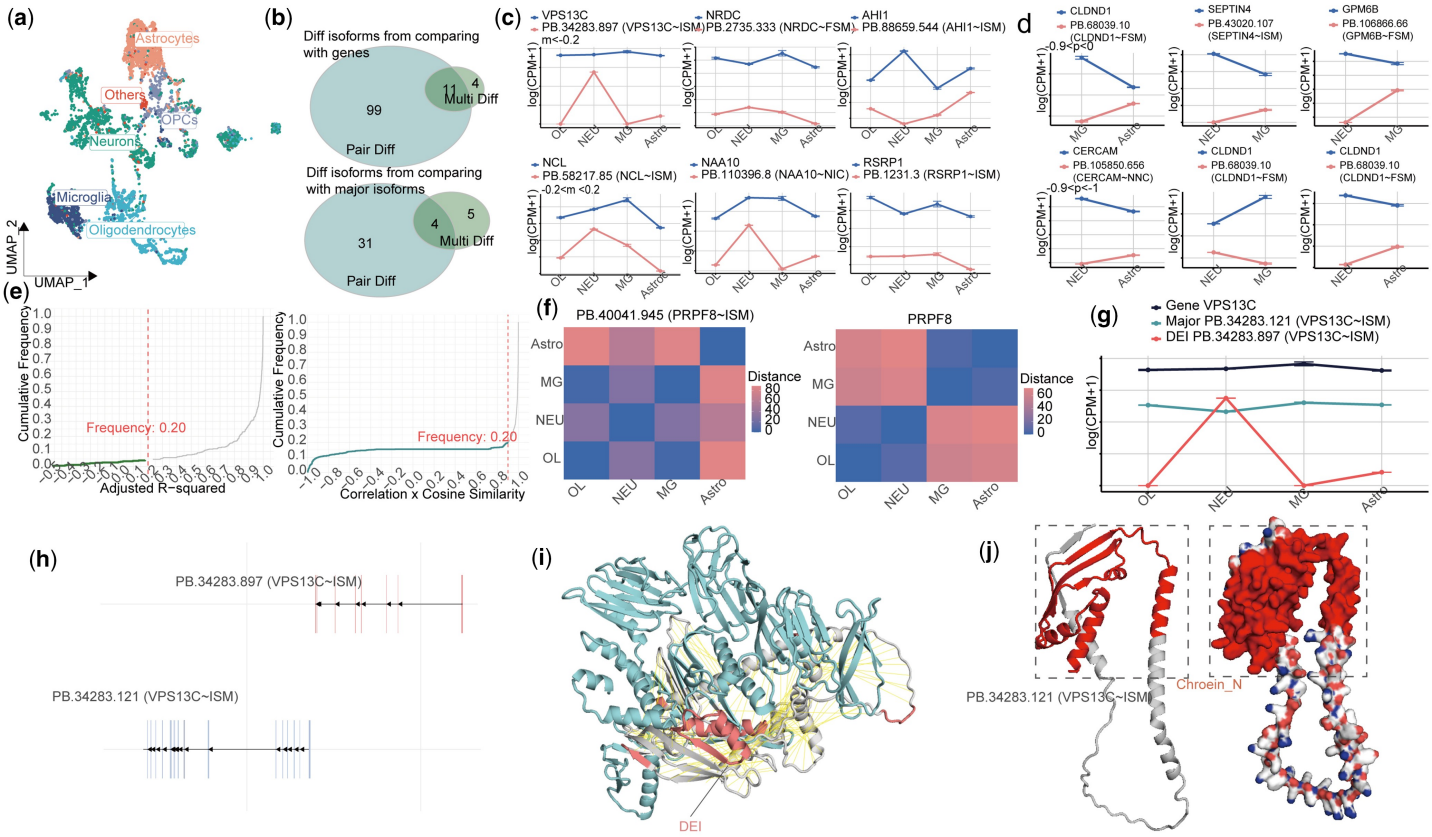
IsoDiffR analysis of human frontal cortex long-read scRNA-seq data. (a) Single-cell representation of the frontal cortex dataset shown by UMAP (left), dot plot (middle), and heatmap (right). (b) Set relationships of pair-DEIs and multi-DEIs obtained by comparing isoforms with their corresponding genes (top) or major isoforms (bottom). (c) Representative expression patterns of three multi-DEIs selected from two adj. *R*^2^ ranges across cell types. (d) Line plots of three pair-DEIs from two correlation × cosine similarity ranges across cell types. (e) Cumulative distributions of adj. *R*^2^ and correlation × cosine similarity for all isoforms. (f) Euclidean distance heatmaps of expression vectors for a representative multi-DEI (left) and its corresponding gene (right). (g) Distinct expression pattern of a *VPS13C*-related DEI. (h) Expression comparison between this DEI (top) and the corresponding major isoform (bottom). (i) Structural comparison of DEI and major isoform proteins, highlighting isoform-specific structural regions (solid fill for DEI-specific region, hatched fill for major isoform-specific region). (j) Ribbon (left) and surface (right) representations of the DEI protein, with the unique Chorein_N domain indicated by an arrow.

Line plot visualization of isoforms uniquely identified by IsoDiffR, IsoformSwitchAnalyzeR, and shared isoforms revealed that DEIs identified by IsoDiffR displayed substantial deviations from gene-level expression, whereas isoforms unique to IsoformSwitchAnalyzeR closely mirrored gene trends (Fig. 3d, Figs 3–6, available as supplementary data at *Bioinformatics* online). These results demonstrate that IsoDiffR provides a more sensitive and nuanced detection of isoform-level differential expression between cell types.

We conducted a comprehensive analysis of the results generated by IsoDiffR, categorizing the identified DEIs into two main groups: isoforms with expression patterns distinct from their corresponding genes, and isoforms with expression patterns distinct from their major isoforms. Each group was further subdivided into multi-DEIs and pair-DEIs (Fig. 3e). Notably, the number of pair-DEIs was significantly higher than that of multi-DEIs, with most multi-DEIs also represented within the pair-DEIs category. Only two multi-DEIs, identified through gene-level comparisons, were not included in the pair-DEIs. We visualized the expression patterns of these two multi-DEIs and their corresponding genes using line plots (Fig. 3f). The results showed that, although these isoforms did not exhibit significant expression differences in pairwise cell type comparisons between cell types, their overall expression trends were notably distinct from those of their corresponding genes.

### 3.4 System-wide analysis of real long-read scRNA-seq datasets reveals biologically meaningful isoform dynamics

To evaluate IsoDiffR in real biological contexts, we applied it to long-read single-cell and single-nucleus RNA-seq datasets. IsoDiffR identifies DEIs exhibiting expression patterns distinct from their corresponding genes or major isoforms. Default thresholds were applied: adj. *R*^2^ < 0.2 for multi-DEIs and correlation × cosine similarity < 0.9 for pair-DEIs (Figs 7a and 11a, available as supplementary data at *Bioinformatics* online). These thresholds effectively separated isoforms with unique expression dynamics from those closely mirroring gene-level patterns. Expression divergences were assessed via line plots, cumulative distributions, and Euclidean distance matrix heatmaps.

#### 3.4.1 *Macaca fascicularis* corneal limbus

Applying IsoDiffR to the corneal limbus dataset, we identified 107 DEIs relative to genes and 80 relative to major isoforms, with minimal overlap between multi- and pair-DEIs (Fig. 4a). A subset of DEIs stratified by threshold intervals was visualized using line plots (Fig. 4b and c) and Figs 8 and 9, available as supplementary data at *Bioinformatics* online. These analyses revealed that DEIs exhibited expression patterns clearly distinct from corresponding genes, while non-DEIs largely mirrored gene expression. Cumulative distribution plots confirmed that multi-DEIs with adj. *R*^2^ < 0.2 accounted for ≈9% of total isoforms, and pair-DEIs clustered near correlation × cosine similarity ≈1 for non-DEIs (Fig. 4d). Cosine similarity distributions stratified by Pearson correlation sign highlighted that discordant expression changes (negative correlations) contributed predominantly to pair-DEIs (Fig. 7b, available as supplementary data at *Bioinformatics* online). Euclidean distance matrix heatmaps further illustrated the divergence in expression patterns between DEIs and their corresponding genes or major isoforms (Fig. 4e), providing an intuitive and quantitative view of isoform-specific regulation.

Functional analysis of multi-DEIs in the *Macaca fascicularis* corneal limbus revealed enrichment in biologically relevant pathways, most notably the Wnt signaling pathway, which plays a central role in regulating corneal limbal stem cell proliferation and differentiation (Fig. 7c, available as supplementary data at *Bioinformatics* online) (Nakatsu *et al.* 2011, Hamilton *et al.* 2016, Wang *et al.* 2024). Within this pathway, *KLF4* functions as a central transcriptional regulator critical for maintaining epithelial identity and controlling stem cell fate(Swamynathan *et al.* 2008, Delp *et al.* 2015, Kim *et al.* 2015, Tiwari *et al.* 2017, Tiwari *et al.* 2019, Li *et al.* 2021, Borisova *et al.* 2022, Li *et al.* 2022, He *et al.* 2023, Swamynathan and Swamynathan 2023, Li *et al.* 2024). The DEI “PB.17036.10 (*KLF4*∼NNC)” emerged as particularly noteworthy, exhibiting significant down-regulation in corneal epithelial cells relative to its corresponding gene and major isoform (Fig. 4f). Structural analysis revealed substantial differences between this DEI and both the annotated transcript and major isoform (Fig. 4g, Fig. 7d, available as supplementary data at *Bioinformatics* online), including unique domain compositions such as KLF4_N, SH3, TAD, and PEST motifs (Table 4, available as supplementary data at *Bioinformatics* online). AlphaFold3 predictions confirmed isoform-specific 3D structures (Fig. 4h, available as supplementary data at *Bioinformatics* online), and STRING-based interaction analysis identified selective binding of the DEI to transcriptional co-activators EP300 and CREBBP (Song *et al.* 2002) (Fig. 4i, Fig. 7e, available as supplementary data at *Bioinformatics* online), supporting functional specificity. These interactions likely facilitate chromatin acetylation and transcriptional regulation, while sequestering CBP/p300 from NF-κB to modulate inflammatory responses (Allen *et al.* 2010). Collectively, these results indicate that the *KLF4* DEI exhibits distinct expression, structural, and functional characteristics, highlighting its potential role in corneal epithelial biology beyond that of the canonical isoform.

#### 3.4.2 Human frontal cortex

To extend IsoDiffR applications beyond the cornea, we analyzed a long-read single-nucleus RNA-seq dataset from the human frontal cortex, identifying six major cell types through clustering and annotation (Fig. 5a, Fig. 10, available as supplementary data at *Bioinformatics* online). Focusing on the four most abundant cell types—Neurons (NEU), Oligodendrocytes (OL), Astrocytes (Astro), and Microglia (MG)—we investigated Alzheimer’s disease (AD)-associated splicing alterations. Due to the sparsity of this dataset, the “min.pct” threshold was reduced from 0.25 to 0.1, yielding 114 DEIs relative to genes and 40 DEIs relative to major isoforms (Fig. 5b, Fig. 11a, available as supplementary data at *Bioinformatics*  online, Table 5, available as supplementary data at *Bioinformatics* online). Line plots of representative DEIs across multiple or pairwise cell-type comparisons confirmed distinct isoform expression patterns, diverging from corresponding genes (Fig. 5c and d, Figs 12 and 13, available as supplementary data at *Bioinformatics* online). The cumulative distribution of adj. *R*^2^ and correlation × cosine similarity values mirrored observations in the *Macaca fascicularis* corneal limbus dataset: multicell-type DEIs sharply increased near 1, while pairwise DEIs largely clustered near 1, indicating that most isoforms resemble their gene or major isoform expression patterns (Fig. 5e, Fig. 11b, available as supplementary data at *Bioinformatics* online). Euclidean distance heatmaps further highlighted the divergence in expression patterns (Fig. 5f).

KEGG pathway analysis of 15 multi-DEIs revealed significant enrichment in the “response to insulin” pathway, implicating insulin signaling in synaptic neurotransmission and glial metabolism, processes known to influence neurodegeneration and AD pathology (Kolehmainen *et al.* 2003, Pedersen and Flynn 2004, Fehm *et al.* 2006, Mizuno *et al.* 2007, Stanley *et al.* 2016, Amarie *et al.* 2017, Arnold *et al.* 2018, Ferreira *et al.* 2018, Sedzikowska and Szablewski 2021, Smolders *et al.* 2021) (Fig. 11c). Among these, the *VPS13C* DEI “PB.34283.897(*VPS13C*∼ISM)” displayed cell-type-specific silencing in OL and MG, while being highly expressed in NEU, in contrast to its gene and major isoform “PB.34283.121(*VPS13C*∼ISM)” (Fig. 5g). Transcript structure comparison revealed no genomic overlap (Fig. 5h), and amino acid sequence analysis indicated substantial differences in length and alignment (Fig. 11d, available as supplementary data at *Bioinformatics* online).

AlphaFold3-based 3D modeling demonstrated limited structural alignment between the DEI and major isoform proteins, each exhibiting unique conformational regions including α-helices, β-sheets, and loops (Fig. 5i). Domain analysis via the NCBI CDD database revealed that the major isoform aligns with the VPS13 family, whereas the DEI contains a Chorein_N domain, a leucine zipper motif critical for lipid transport and vesicular trafficking (Kumar *et al.* 2018, Lees and Reinisch 2020a,b) (Table 6, available as supplementary data at *Bioinformatics* online). Structural visualization of the DEI protein highlighted the Chorein_N region (first 154 amino acids) forming a hydrophobic surface conducive to intracellular lipid exchange (Fig. 5j), supporting isoform-specific functional roles distinct from the canonical isoform (Kumar *et al.* 2018, Lees and Reinisch 2020a,b).

## 4 Discussion

The rapid development of scRNA-seq has greatly advanced our understanding of gene expression and cellular heterogeneity, yet isoform-level analysis in long-read single-cell datasets remains insufficiently explored. Existing methods largely focus on exon usage or splicing ratios, leaving a gap in the comprehensive characterization of full-length RNA isoforms. To address this, we developed IsoDiffR, a dedicated tool for long-read scRNA-seq that uses linear regression models to identify isoforms whose expression patterns significantly deviate from those of their corresponding genes or major isoforms across two or more cell types. Beyond detection, IsoDiffR facilitates downstream exploration of these isoforms through analyses of expression dynamics, pathway enrichment, protein structure prediction, and binding interactions, enabling deeper insight into their potential functions and regulatory roles.

In benchmarking using pseudo-bulk data simulated from long-read scRNA-seq, IsoDiffR showed superior sensitivity and precision to IsoformSwitchAnalyzeR in pair-DEI detection. At the pseudo-bulk level, IsoDiffR also excelled in multi-DEI analysis, demonstrating robustness across different comparison settings. Importantly, IsoDiffR is well suited to true scRNA-seq data, where it reliably identifies DEIs at single-cell resolution and reveals substantial isoform-level heterogeneity underlying complex cellular states.

We further applied IsoDiffR to long-read datasets from the *Macaca fascicularis* corneal limbus and the human frontal cortex. In both tissues, IsoDiffR identified DEIs whose expression patterns diverged markedly from those of their genes or major isoforms in pairwise and multigroup comparisons. Subsequent analyses—including amino acid sequence alignment, transcript structure visualization, and protein structural and functional predictions—highlighted distinct isoform features not captured at the gene level. KEGG pathway enrichment and conserved domain annotation further underscored the biological relevance of these DEIs, revealing specialized functional roles masked in gene-level analyses.

Overall, IsoDiffR provides a robust and comprehensive framework for differential isoform analysis in long-read scRNA-seq data. By accurately detecting DEIs and supporting extensive functional characterization, IsoDiffR uncovers biologically meaningful isoform-specific mechanisms that enhance our understanding of transcriptomic complexity at single-cell resolution and expand the methodological landscape for isoform-focused scRNA-seq research.

## Supplementary Material

btaf664_Supplementary_Data
