# Supplementary material for: Analyzing cell-type-specific isoform expression using IsoDiffR and long-read single-cell RNA sequencing
Source: Bioinformatics. 2025 Dec 16;42(1):btaf664. doi: 10.1093/bioinformatics/btaf664 (PMC12822774; doi:10.1093/bioinformatics/btaf664)
Supplement: btaf664_Supplementary_Data [file btaf664_supplementary_data.pdf]

Supplementary Information

TABLE OF CONTENTS

Supplementary Figures

Supplementary Fig. S1: Empirical FDR and performance of DEIs detection.

Supplementary Fig. S2: Single-cell atlas of long-read scRNA-seq data from the Macaca fascicularis corneal limbus.

Supplementary Fig. S3: Expression patterns of pair-DEIs compared to genes.

Supplementary Fig. S4-5: Expression patterns of switch isoforms obtained from IsoformSwitchAnalyzeR.

Supplementary Fig. S6: Expression patterns of shared isoforms between pair-DEI and Switch.

Supplementary Fig. S7: Application of IsoDiffR on the Macaca fascicularis corneal limbus long-read scRNA-seq data.

Supplementary Fig. S8: Expression patterns of isoforms in different adj R^2 value ranges when comparing across multiple cell types in the Macaca fascicularis long-read scRNA-seq data.

Supplementary Fig. S9: Expression patterns of isoforms in different ranges of the product of pearson correlation coefficient and cosine similarity in the comparison between two cell types in the Macaca fascicularis corneal limbus long-read scRNA-seq data.

Supplementary Fig. S10: Single-cell atlas of human frontal cortex based on long-read scRNA-seq data.

Supplementary Fig. S11: Application of DiffIsoR on long-read scRNA-seq data from human frontal cortex.

Supplementary Fig. S12: Expression patterns of isoforms in different adj R^2 value ranges in the long-read scRNA-seq data of human brain tissue slices when comparing more than two cell types.

Supplementary Fig. S13: Expression patterns of isoforms in different value

ranges of the product of pearson correlation coefficient and cosine similarity in the long-read scRNA-seq data of human frontal cortex when comparing two cell types.

Supplementary Tables

Supplementary Table1. Evaluation of IsoDiffR and IsoformSwitchAnalyzeR Using Simulated Bulk Data.

Supplementary Table2. Evaluation of IsoDiffR and IsoSwitch Using Simulated Single-cell RNA-seq Data.

Supplementary Table3. Shared isoforms between switch isoform and DEI.

Supplementary Table 4. CDD comparison of the major isoform and DEI amino acid sequences.

Supplementary Table 5. Threshold of “min.pct” and No. of DEIs.

Supplementary Table 6. CDD comparison of the major isoform and DEI amino acid sequences.

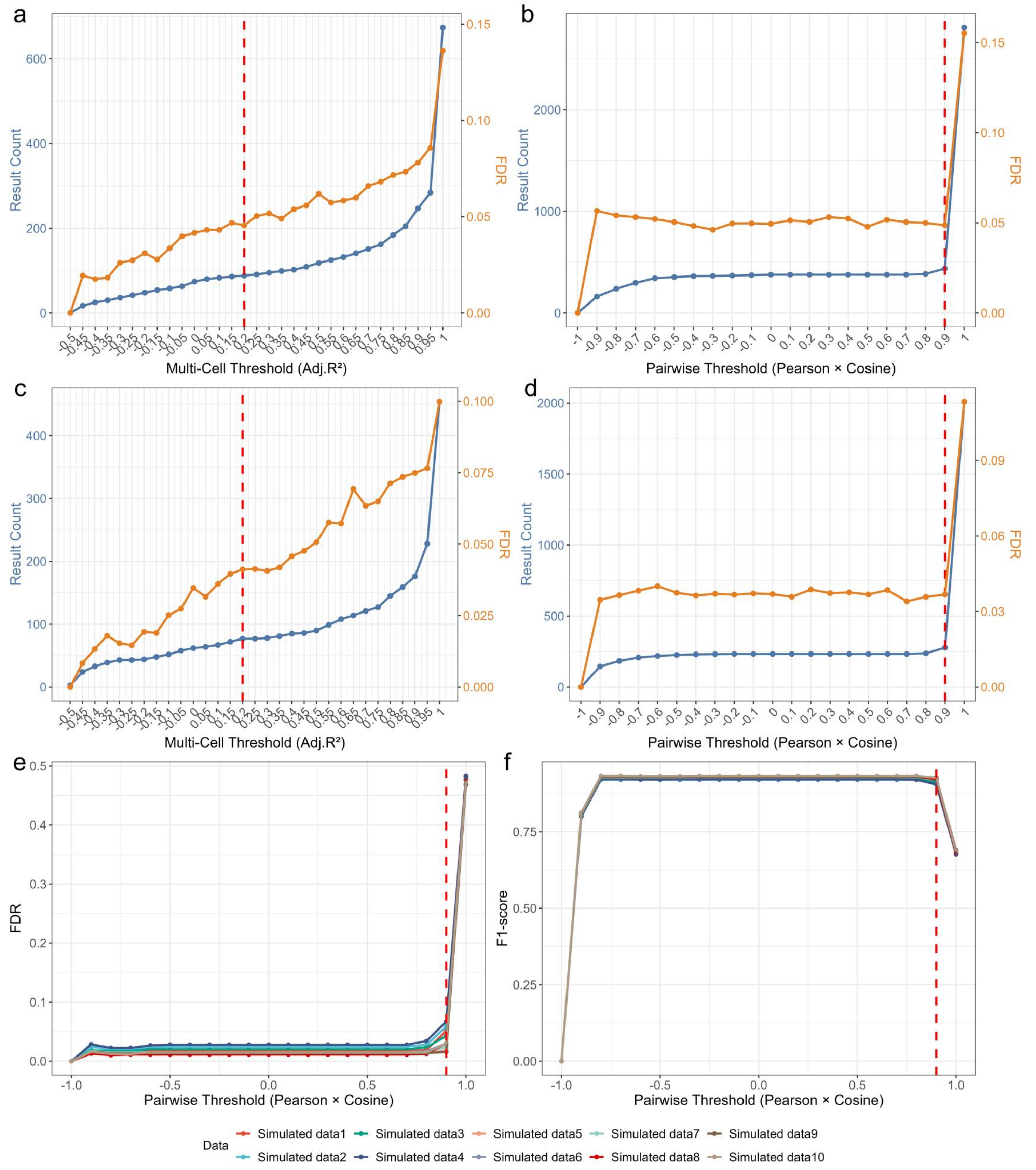

Supplementary Fig. S1: Empirical FDR and performance of DEIs detection. (a-d) Number of identified isoforms (left y-axis) and empirical FDR (right y-axis) for multi- (a, c) and pair-DEIs (b, d) in two real datasets; red dashed line indicates default thresholds. (e-f) Empirical FDR (e) and F1 score (f) for pair-DEIs in simulated datasets with varying sequencing depth or cell numbers; red dashed line indicates default thresholds.

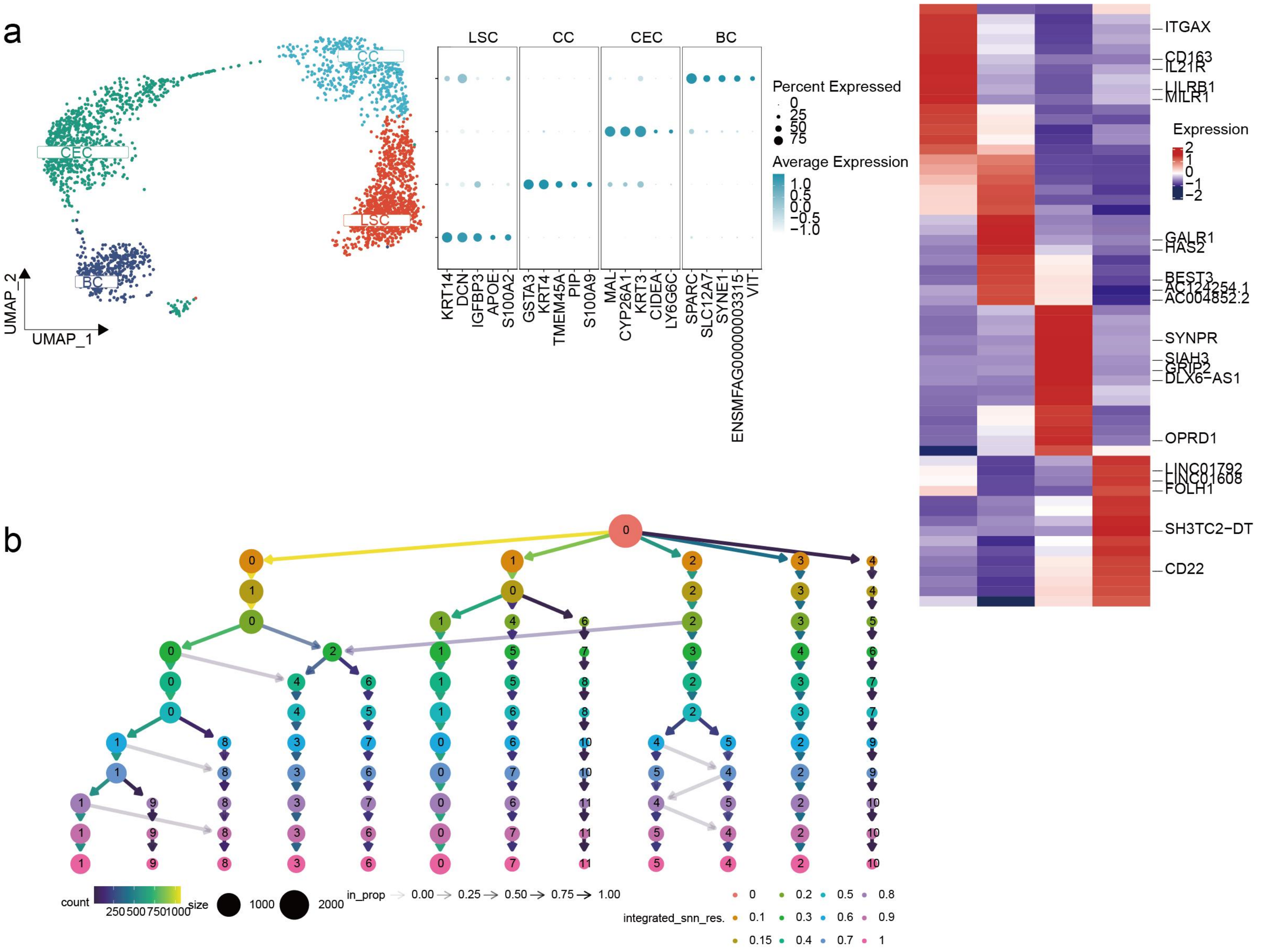

Supplementary Fig. S2: Single-cell atlas of long-read scRNA-seq data from the *Macaca fascicularis* corneal limbus. (a) Single-cell atlas of *Macaca fascicularis* corneal limbus long-read scRNA-seq sequencing data at the gene level, including UMAP(left panel), dot plot(middle panel), and heatmap(right panel). (b) Clustree results of cell clustering.

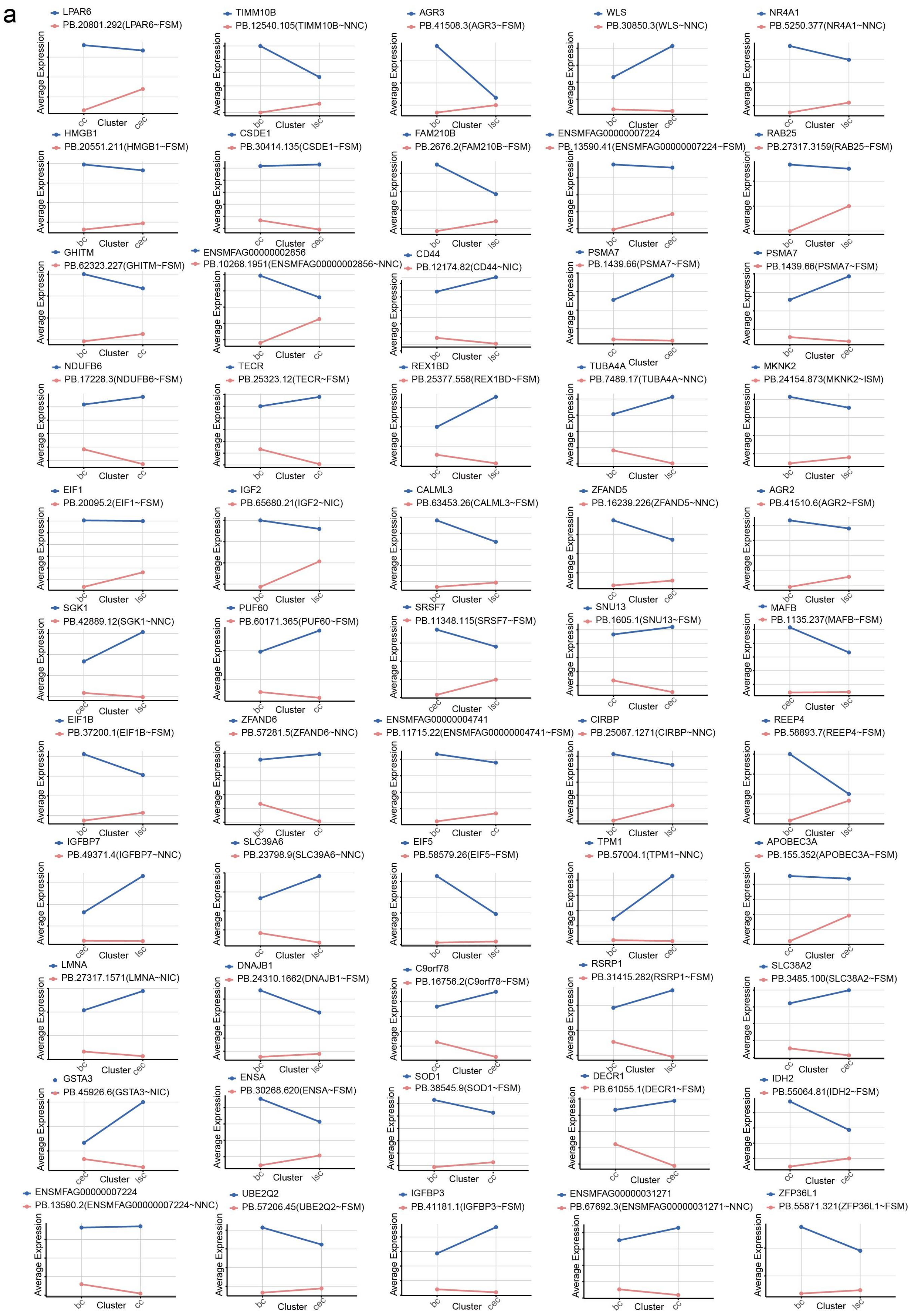

Supplementary Fig. S3: Expression patterns of pair-DEIs compared to genes. (a) Line plots comparing the expression patterns of pair-DEI-specific isoforms with gene-level expression across cell types, relative to the switch isoform.

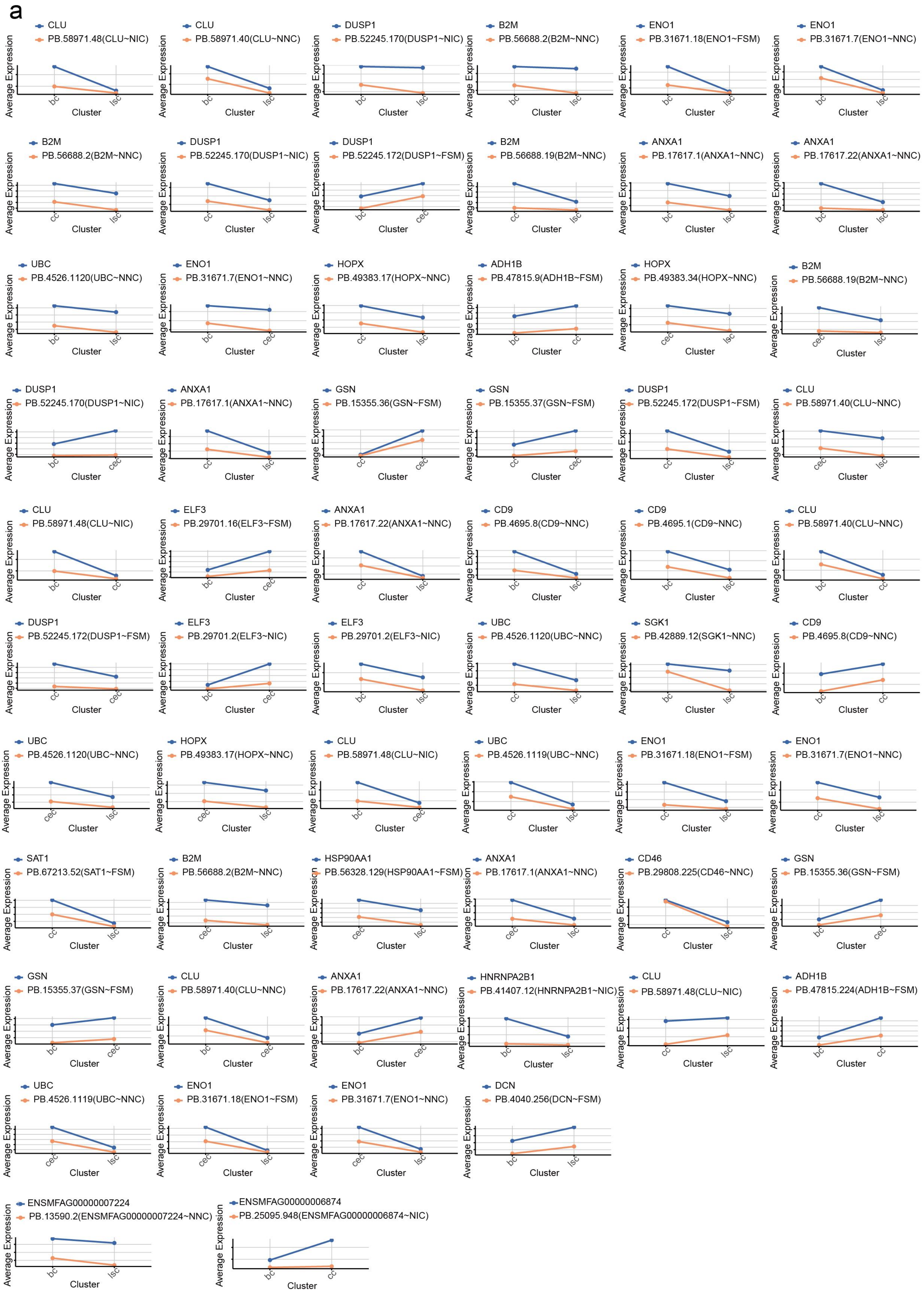
a
Average Expression
Cluster
CLU
PB.58971.48(CLU~NIC)
bc lsc
CLU
PB.58971.40(CLU~NNC)
bc lsc
DUSP1
PB.52245.170(DUSP1~NIC)
bc lsc
B2M
PB.56688.2(B2M~NNC)
bc lsc
ENO1
PB.31671.18(ENO1~FSM)
bc lsc
ENO1
PB.31671.7(ENO1~NNC)
bc lsc
B2M
PB.56688.2(B2M~NNC)
cc lsc
DUSP1
PB.52245.170(DUSP1~NIC)
cc lsc
DUSP1
PB.52245.172(DUSP1~FSM)
bc cec
B2M
PB.56688.19(B2M~NNC)
cc lsc
ANXA1
PB.17617.1(ANXA1~NNC)
bc lsc
ANXA1
PB.17617.22(ANXA1~NNC)
bc lsc
UBC
PB.4526.1120(UBC~NNC)
bc lsc
ENO1
PB.31671.7(ENO1~NNC)
bc cec
HOPX
PB.49383.17(HOPX~NNC)
cc lsc
ADH1B
PB.47815.9(ADH1B~FSM)
bc cc
HOPX
PB.49383.34(HOPX~NNC)
cec lsc
B2M
PB.56688.19(B2M~NNC)
cec lsc
DUSP1
PB.52245.170(DUSP1~NIC)
bc cec
ANXA1
PB.17617.1(ANXA1~NNC)
cc lsc
GSN
PB.15355.36(GSN~FSM)
cc cec
GSN
PB.15355.37(GSN~FSM)
cc cec
DUSP1
PB.52245.172(DUSP1~FSM)
cc lsc
CLU
PB.58971.40(CLU~NNC)
cec lsc
CLU
PB.58971.48(CLU~NIC)
bc cc
ELF3
PB.29701.16(ELF3~FSM)
bc cec
ANXA1
PB.17617.22(ANXA1~NNC)
cc lsc
CD9
PB.4695.8(CD9~NNC)
bc lsc
CD9
PB.4695.1(CD9~NNC)
bc lsc
CLU
PB.58971.40(CLU~NNC)
bc cc
DUSP1
PB.52245.172(DUSP1~FSM)
cc cec
ELF3
PB.29701.2(ELF3~NIC)
bc cec
ELF3
PB.29701.2(ELF3~NIC)
bc lsc
UBC
PB.4526.1120(UBC~NNC)
cc lsc
SGK1
PB.42889.12(SGK1~NNC)
bc lsc
CD9
PB.4695.8(CD9~NNC)
bc cc
UBC
PB.4526.1120(UBC~NNC)
cec lsc
HOPX
PB.49383.17(HOPX~NNC)
cec lsc
CLU
PB.58971.48(CLU~NIC)
bc cec
UBC
PB.4526.1119(UBC~NNC)
cc lsc
ENO1
PB.31671.18(ENO1~FSM)
cc lsc
ENO1
PB.31671.7(ENO1~NNC)
cc lsc
SAT1
PB.67213.52(SAT1~FSM)
cc lsc
B2M
PB.56688.2(B2M~NNC)
cec lsc
HSP90AA1
PB.56328.129(HSP90AA1~FSM)
cec lsc
ANXA1
PB.17617.1(ANXA1~NNC)
cec lsc
CD46
PB.29808.225(CD46~NNC)
cc lsc
GSN
PB.15355.36(GSN~FSM)
bc cec
GSN
PB.15355.37(GSN~FSM)
bc cec
CLU
PB.58971.40(CLU~NNC)
bc cec
ANXA1
PB.17617.22(ANXA1~NNC)
bc cec
HNRNPA2B1
PB.41407.12(HNRNPA2B1~NIC)
bc lsc
CLU
PB.58971.48(CLU~NIC)
cc lsc
ADH1B
PB.47815.224(ADH1B~FSM)
bc cc
UBC
PB.4526.1119(UBC~NNC)
cec lsc
ENO1
PB.31671.18(ENO1~FSM)
cec lsc
ENO1
PB.31671.7(ENO1~NNC)
cec lsc
DCN
PB.4040.256(DCN~FSM)
bc lsc
ENSMFAG00000007224
PB.13590.2(ENSMFAG00000007224~NNC)
bc lsc
ENSMFAG00000006874
PB.25095.948(ENSMFAG00000006874~NIC)
bc cc

a

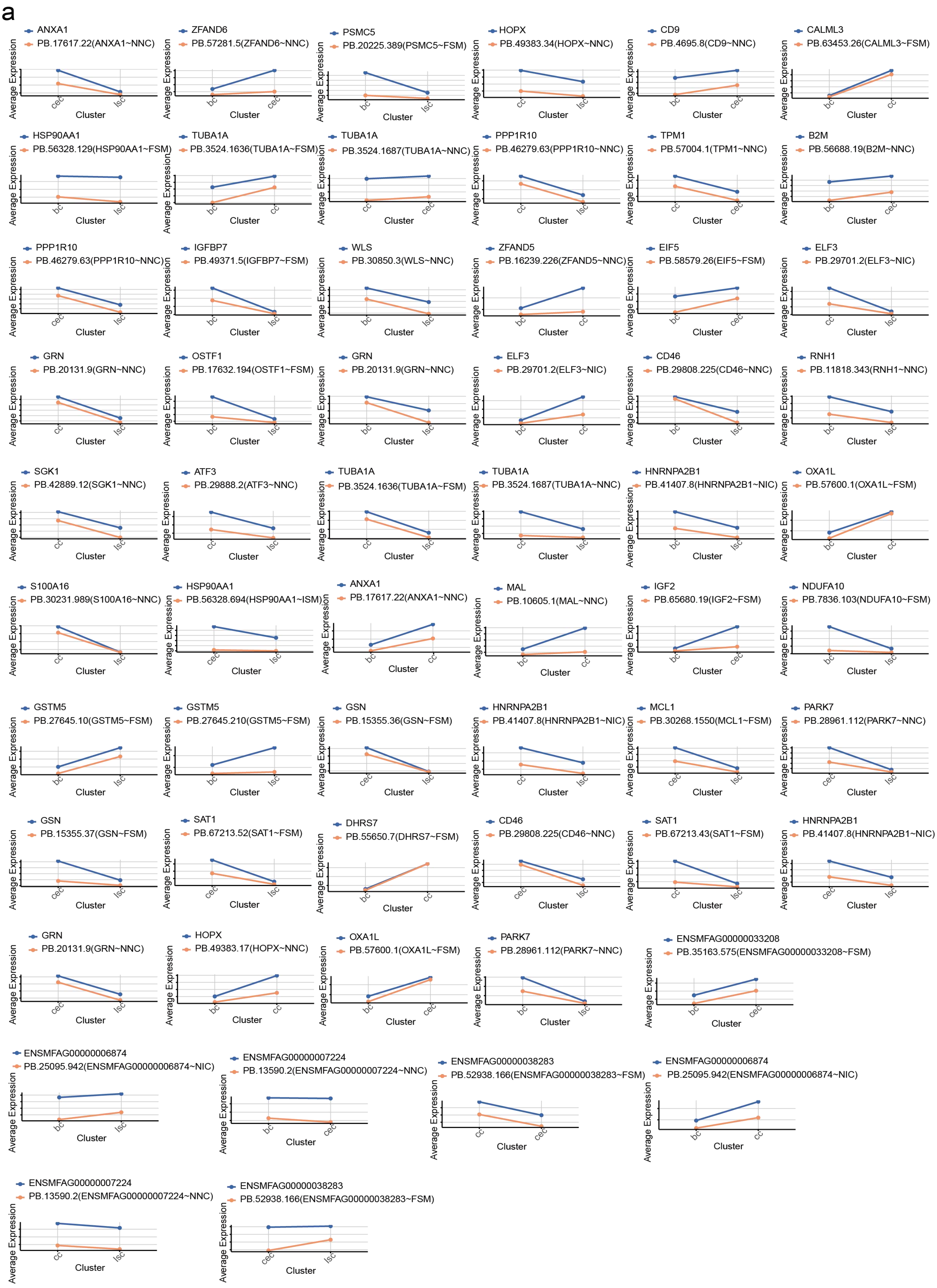

Supplementary Fig. S4-5: Expression patterns of switch isoforms obtained from IsoformSwitchAnalyzeR. (a) Line plots comparing the expression patterns of switch isoform-specific isoforms with gene-level expression across cell types.

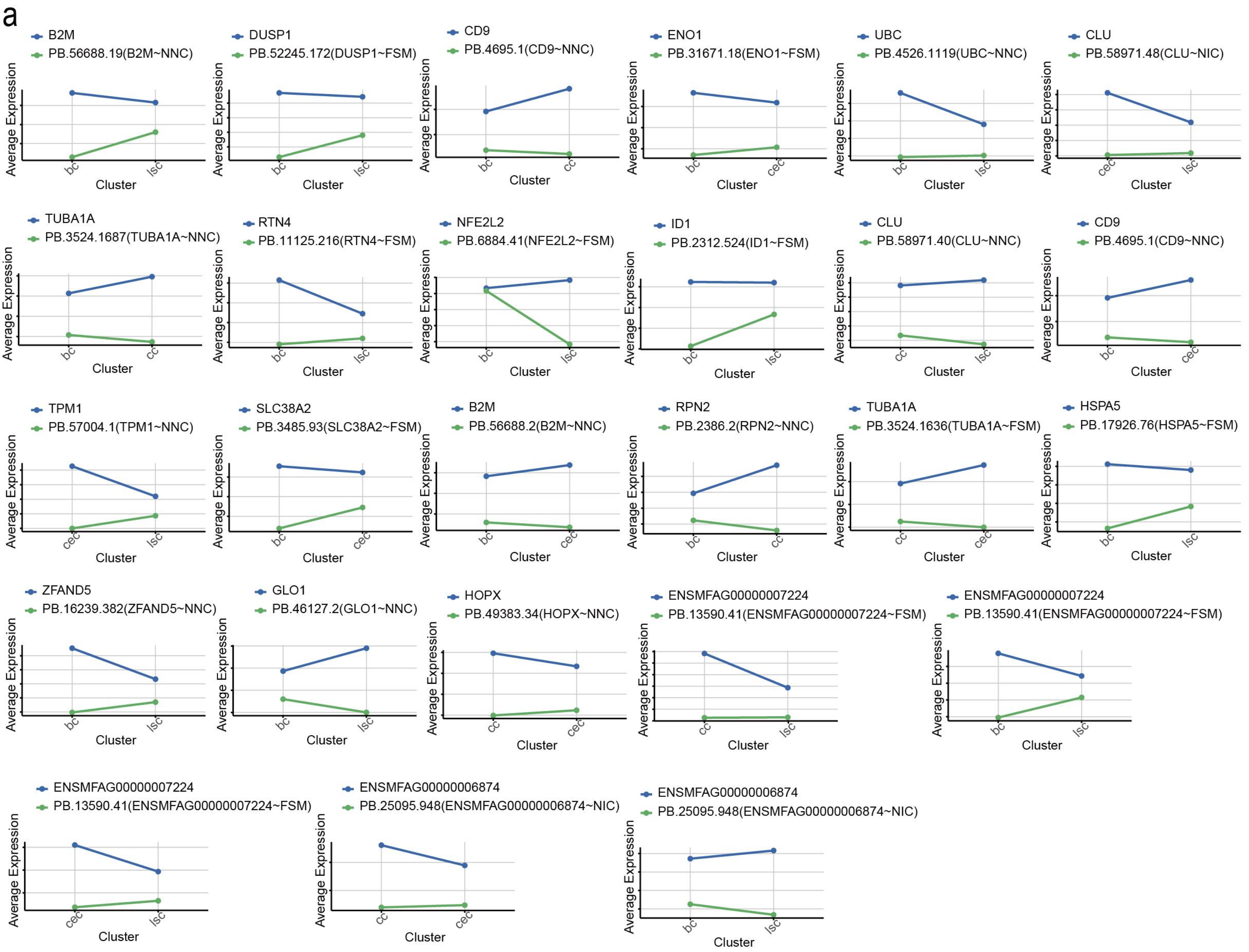

Supplementary Fig. S6: Expression patterns of shared isoforms between pair-DEI and Switch. (a) Line plots comparing the expression patterns of shared isoforms between pair-DEI and Switch with gene-level expression across cell types.

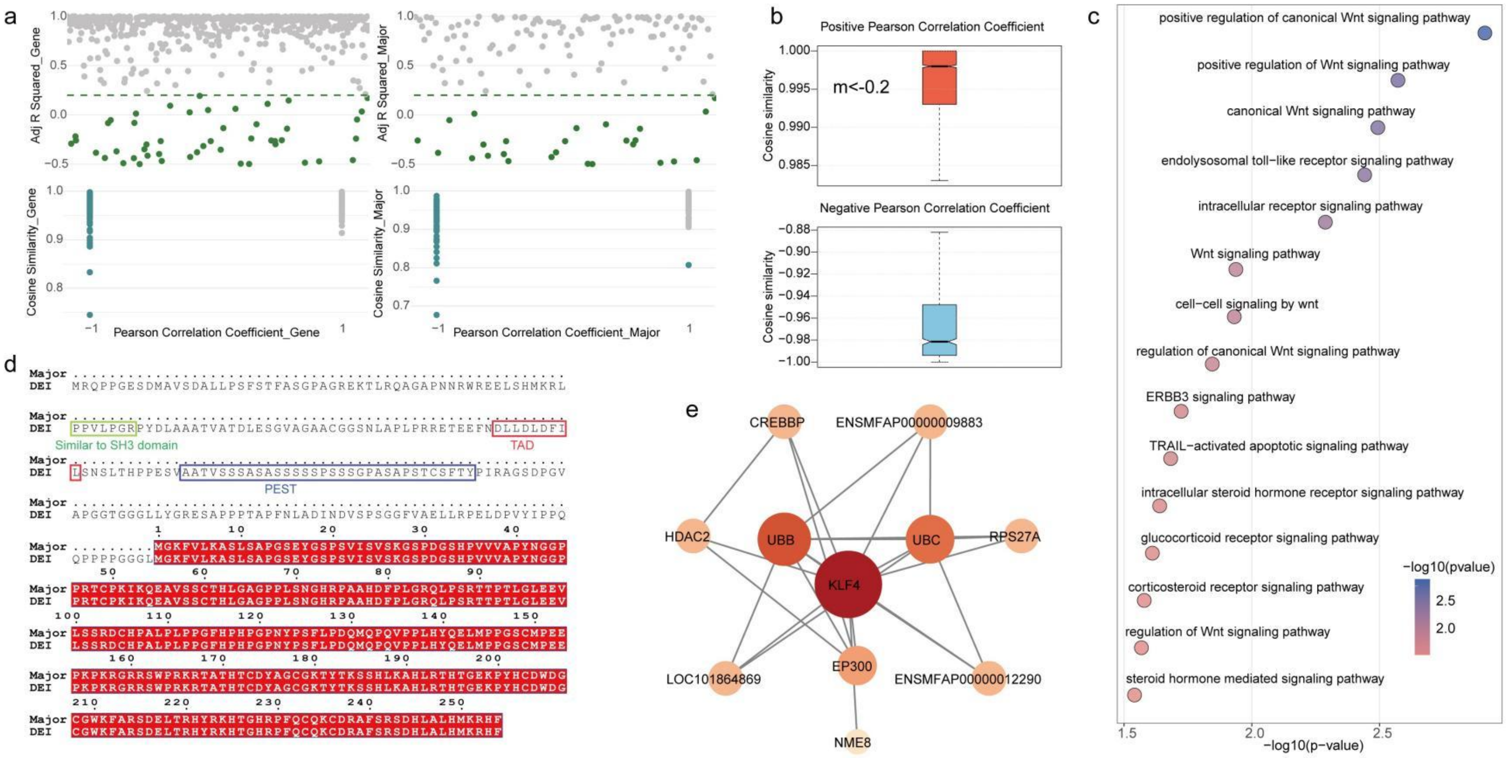

Supplementary Fig. S7: Application of IsoDiffR on the Macaca fascicularis corneal limbus long-read scRNA-seq data. (**a**) Distribution of adj.R^2 values as well as correlation and cosine similarity values obtained by comparing isoforms with Gene and major iso-form in multi-cell type comparisons and pairwise cell type comparisons, respectively. (**b**) Distribution of cosine similarity values when the correlation is positive (top panel) or negative (bottom panel) in pairwise cell type comparisons. (**c**) KEGG pathway map showing the key biological processes involving the multi-DEIs compared with corresponding genes. (**d**) Alignment of the amino acid sequences of DEI and major isoform. (**e**) Interaction gene network of the gene KLF4.

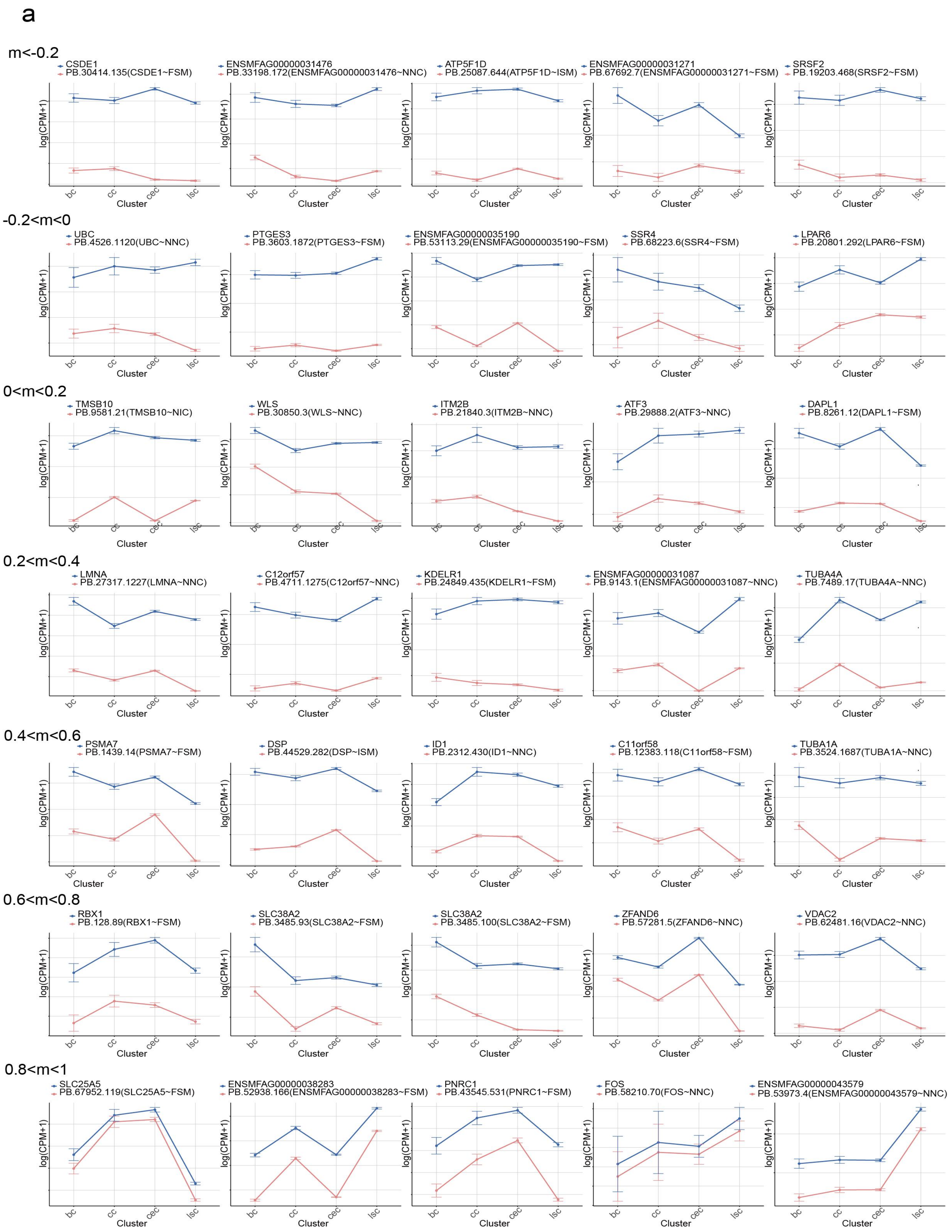

Supplementary Fig. S8: Expression patterns of isoforms in different adj R^2 value ranges when comparing across multiple cell types in the *Macaca fascicularis* long-read scRNA-seq data. (a) Line plots comparing the expression patterns of candidate isoforms from multi-DEI in different adj R^2 value ranges with gene expression.

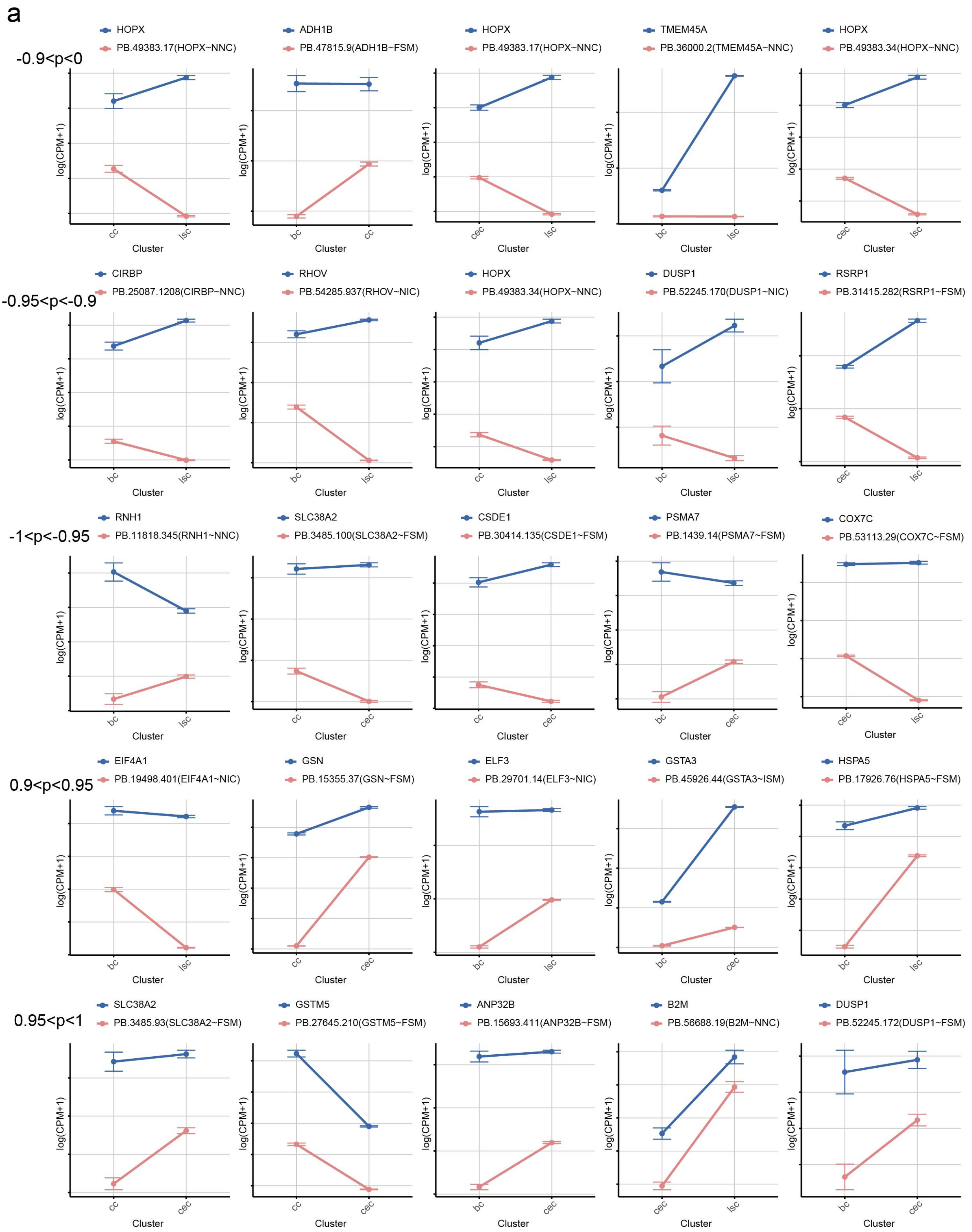

Supplementary Fig. S9: Expression patterns of isoforms in different ranges of the product of pearson correlation coefficient and cosine similarity in the comparison between two cell types in the *Macaca fascicularis* corneal limbus long-read scRNA-seq data. (**a**[1-4]) Line plot comparing the expression patterns of candidate isoforms of pair-DEI with gene expression patterns in various ranges of the product of Pearson Correlation Coefficient and Cosine Similarity.

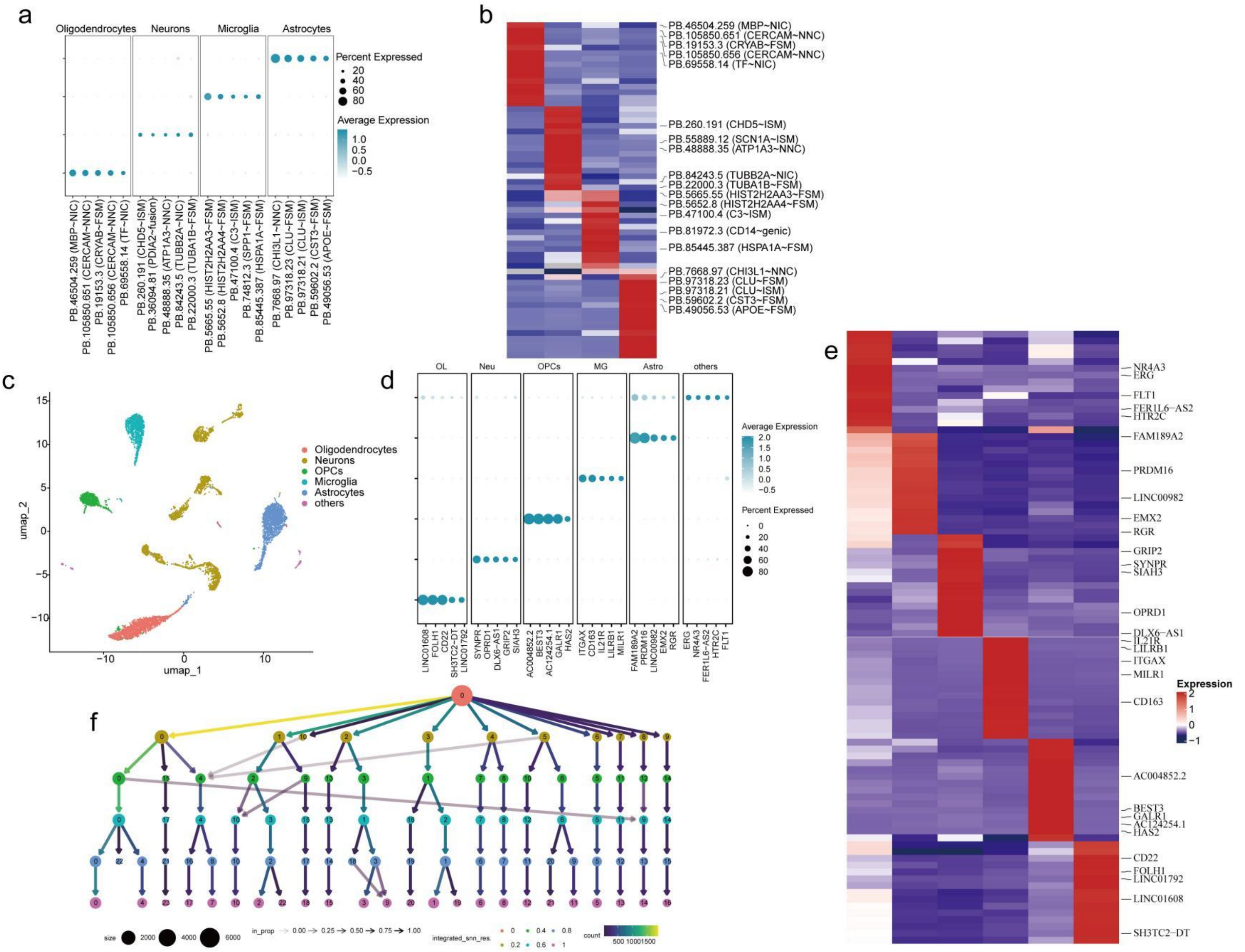

Supplementary Fig. S10: Single-cell atlas of human frontal cortex based on long-read scRNA-seq data. (**a-b**) Single-cell map of the human **frontal** cortex long-read scRNA-seq data, shown as dotplot (left panle) and heatmap (right panle). (**c-e**) Single-cell atlas at the gene level for human frontal cortex, including UMAP, dotplot, and heatmap. (**f**) Clustree result of cell clustering.

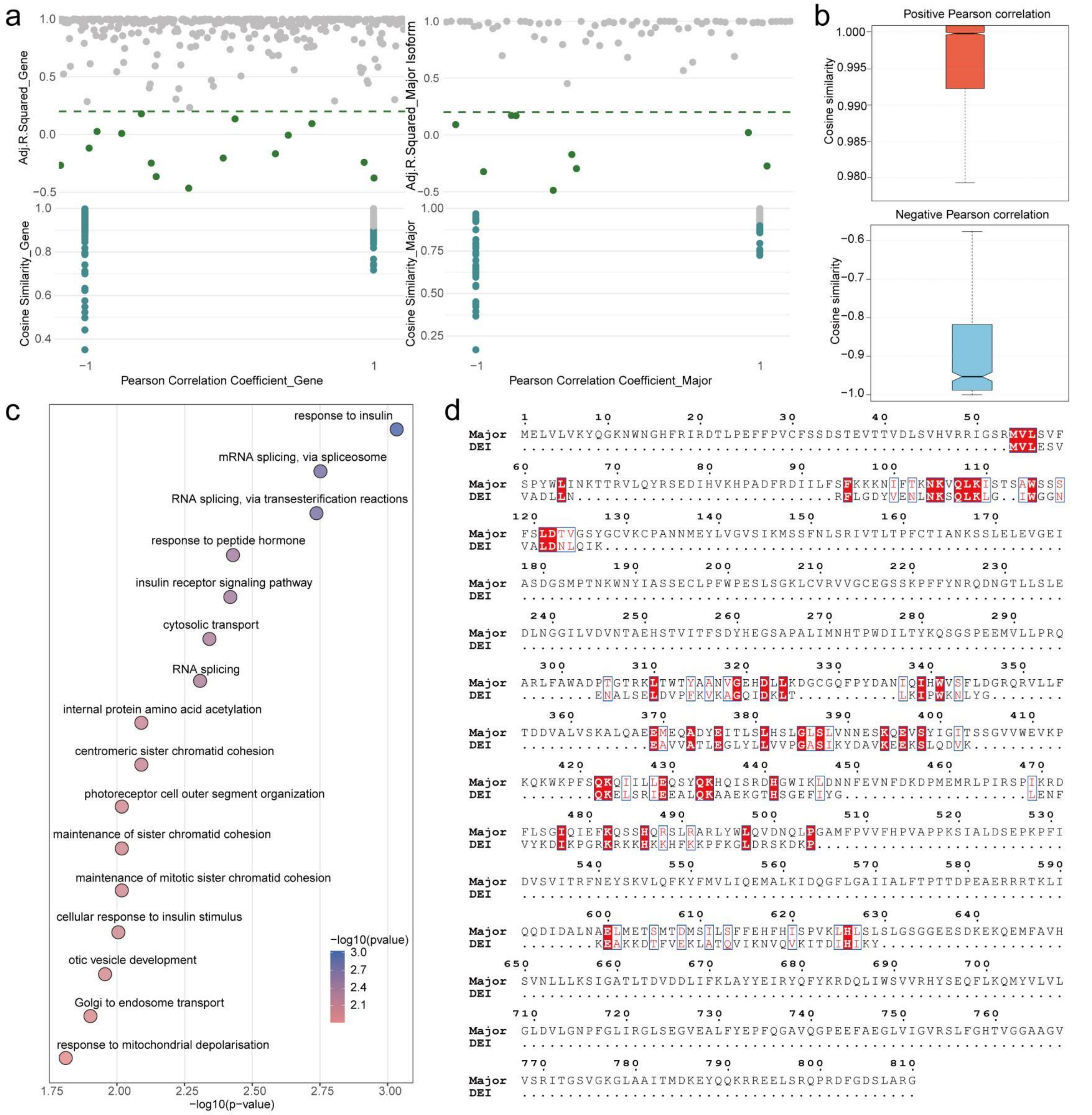

Supplementary Fig. S11: Application of DiffIsoR on long-read scRNA-seq data from human frontal cortex. (**a**) Distribution of adj.R^2 values as well as correlation and cosine similarity values obtained from multi-cell type comparisons between iso-form, Gene, and major isoform. (**b**) Distribution of cosine similarity values when the correlation is positive (top panel) or negative (bottom panel) in pairwise cell type comparisons. (**c**) KEGG pathway map showing the key biological processes involving the multi-DEIs compared with corresponding genes. (**d**) Alignment of the amino acid sequences of DEI and major isoform.

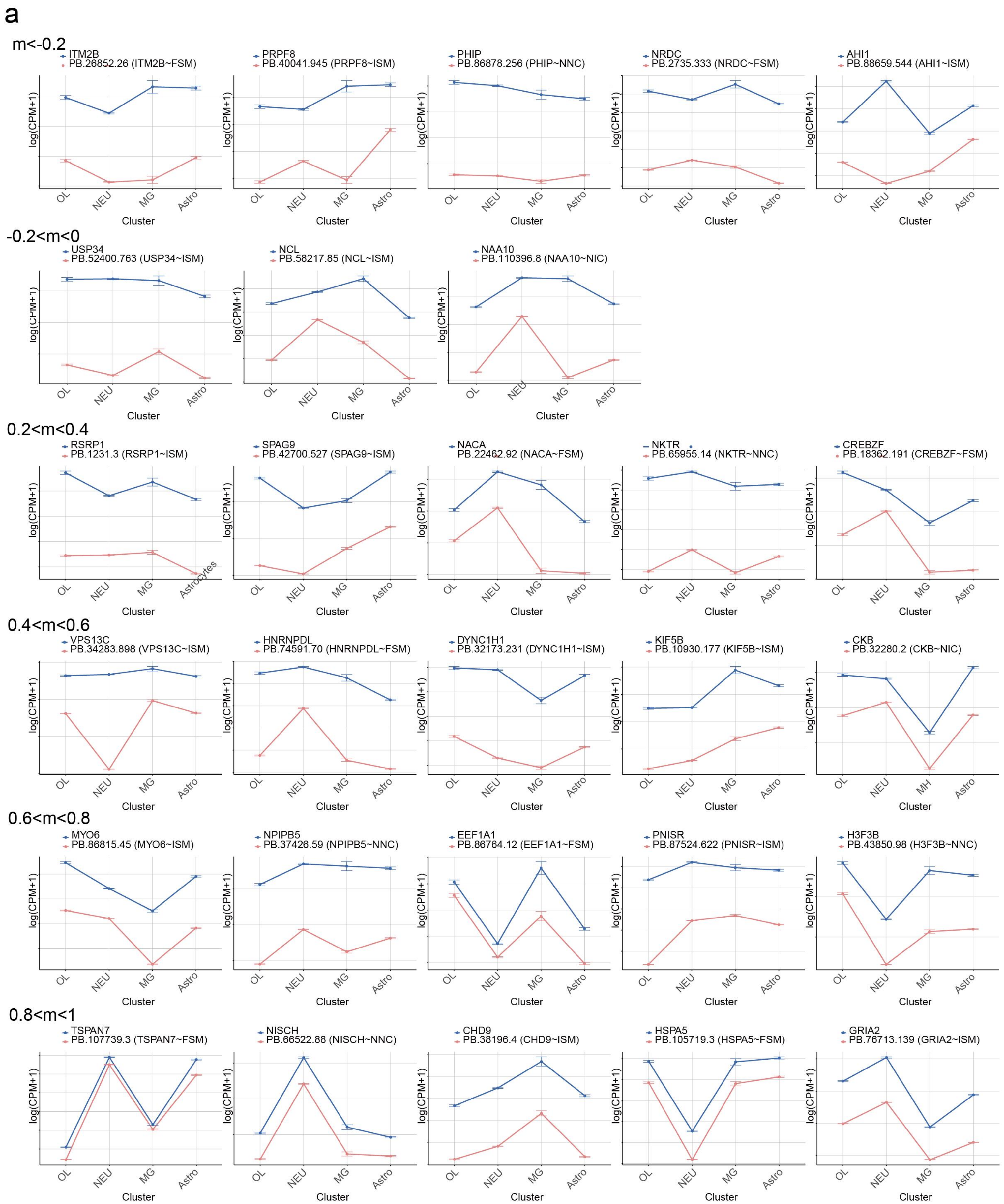

Supplementary Fig. S12: Expression patterns of isoforms in different adj R² value ranges in the long-read scRNA-seq data of human brain **tissue** slices when comparing more than two cell types. (a) Line plot comparing the expression patterns of candidate isoforms from multi-DEI in different adj R² value ranges with gene expression patterns.

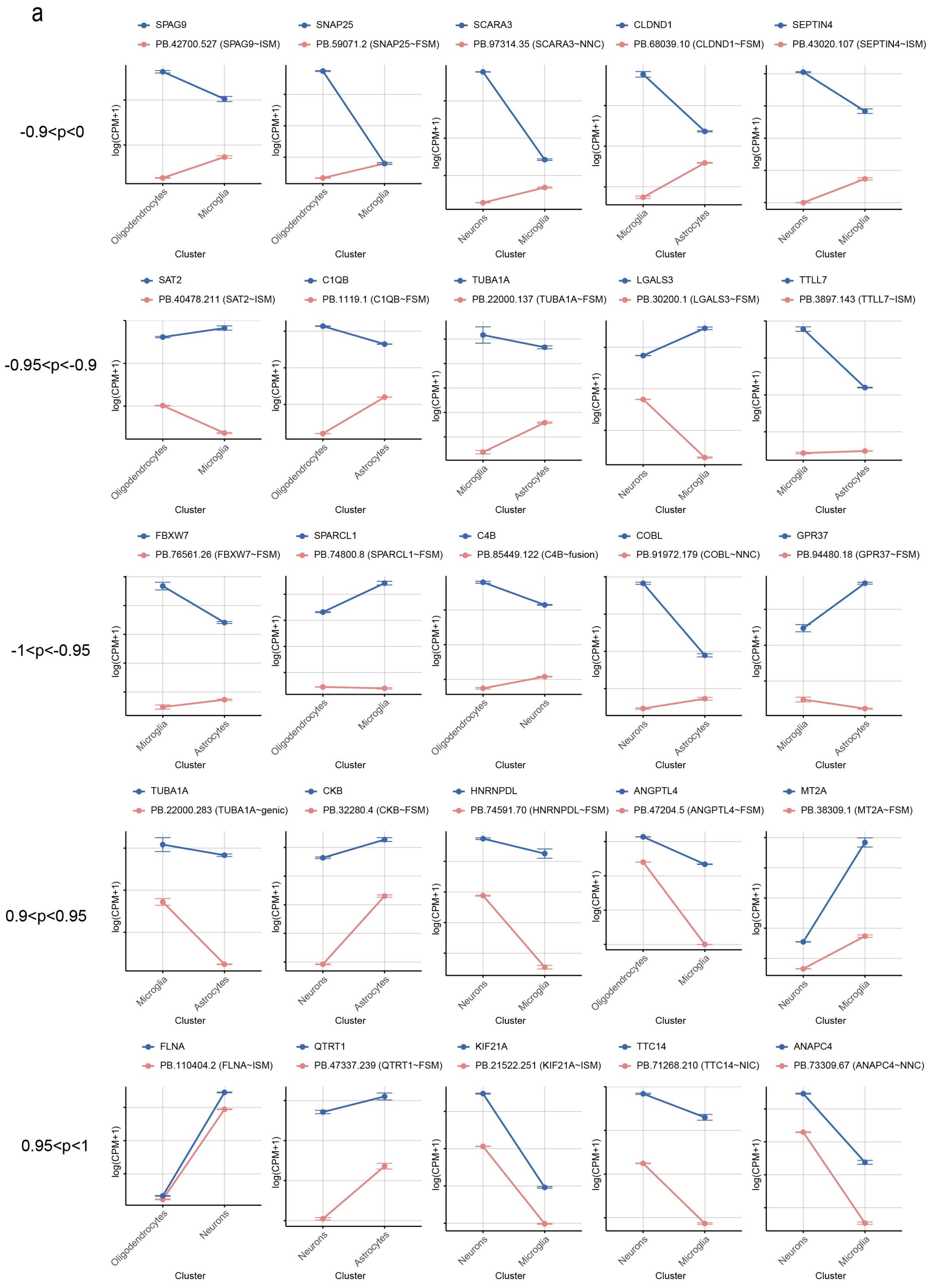

Supplementary Fig. S13: Expression patterns of isoforms in different value ranges of the product of pearson correlation coefficient and cosine similarity in the long-read scRNA-seq data of human frontal cortex when comparing two cell types. (a) Line plot comparing the expression patterns of candidate isoforms from pair-DEI in different value ranges of the product of pearson correlation coefficient and cosine similarity with gene expression patterns.

Supplementary Table 1. Evaluation of IsoDiffR and IsoformSwitchAnalyzeR Using Simulated Bulk Data

		IsoDiffR		IsoformSwitchAnalyzeR	
Depth	Metric	sample1	sample2	sample1	sample2
0.5M	Precision	0.981	0.973	0.529	0.523
	Recall	0.892	0.899	0.982	0.972
	F1-score	0.934	0.935	0.688	0.68
	FDR	0.019	0.027	0.471	0.477
1M	Precision	0.975	0.972	0.526	0.527
	Recall	0.893	0.892	0.983	0.98
	F1-score	0.932	0.931	0.685	0.686
	FDR	0.025	0.028	0.474	0.473
2M	Precision	0.976	0.979	0.529	0.527
	Recall	0.891	0.893	0.983	0.983
	F1-score	0.932	0.934	0.688	0.686
	FDR	0.024	0.024	0.471	0.473

Supplementary Table 2. Evaluation of IsoDiffR and IsoSwitch Using Simulated Single-cell RNA-seq Data

Tool	Depth	Cell Count	Comparison	Precision	Recall	F1-score	FDR
IsoDiffR	0.5M	100	sample1	0.899	0.875	0.887	0.101
			sample2	0.867	0.857	0.862	0.133
		300	sample1	0.893	0.874	0.883	0.107
			sample2	0.838	0.859	0.848	0.162
		1000	sample1	0.881	0.871	0.876	0.119
			sample2	0.844	0.861	0.852	0.156
	1M	100	sample1	0.949	0.873	0.909	0.051
			sample2	0.942	0.874	0.907	0.058
		300	sample1	0.957	0.873	0.913	0.043
			sample2	0.933	0.878	0.905	0.067
		1000	sample1	0.952	0.874	0.911	0.048
			sample2	0.929	0.874	0.901	0.071
	2M	100	sample1	0.982	0.877	0.927	0.018
			sample2	0.970	0.879	0.922	0.030
		300	sample1	0.984	0.877	0.928	0.016
			sample2	0.970	0.886	0.926	0.030
		1000	sample1	0.977	0.877	0.924	0.023
			sample2	0.970	0.878	0.922	0.030
IsoSwitch	0.5M	100	sample1	0.189	0.393	0.255	0.811
			sample2	0.178	0.397	0.246	0.822
		300	sample1	0.086	0.408	0.142	0.914
			sample2	0.094	0.409	0.153	0.906
		1000	sample1	0.043	0.462	0.079	0.957
			sample2	0.032	0.464	0.060	0.968
	1M	100	sample1	0.329	0.380	0.353	0.671
			sample2	0.285	0.378	0.325	0.715
		300	sample1	0.221	0.355	0.273	0.779
			sample2	0.206	0.348	0.259	0.794
		1000	sample1	0.128	0.365	0.190	0.872
			sample2	0.127	0.367	0.189	0.873
	2M	100	sample1	0.463	0.365	0.408	0.537
			sample2	0.429	0.355	0.389	0.571
		300	sample1	0.360	0.346	0.353	0.640
			sample2	0.332	0.342	0.337	0.668
		1000	sample1	0.245	0.316	0.276	0.755
			sample2	0.215	0.320	0.257	0.785

Supplementary Table 3. Shared isoforms between switch isoform and DEI. Isoforms defined as both switch isoforms and pair-DEI isoforms, along with their parameters from the two tools.

isoform	condition	absdIF	Correlation x Cosine similarity
PB.56688.19(B2M~NNC)	LSC_BC	0.224	-0.986
PB.52245.172(DUSP1~FSM)	LSC_BC	0.219	-0.98
PB.13590.41(ENSMFAG00000007224~FSM)	LSC_BC	0.251	-0.984
PB.31671.18(ENO1~FSM)	CEC_BC	0.129	-0.989
PB.4526.1119(UBC~NNC)	LSC_BC	0.165	-0.992
PB.58971.48(CLU~NIC)	LSC_CEC	0.176	-0.989
PB.4695.1(CD9~NNC)	CC_BC	0.15	-0.982
PB.11125.216(RTN4~FSM)	LSC_BC	0.272	-0.923
PB.6884.41(NFE2L2~FSM)	LSC_BC	0.198	-0.994
PB.13590.41(ENSMFAG00000007224~FSM)	LSC_CEC	0.138	-0.996
PB.58971.40(CLU~NNC)	LSC_CC	0.124	-0.983
PB.4695.1(CD9~NNC)	CEC_BC	0.142	-0.984
PB.3524.1687(TUBA1A~NNC)	CC_BC	0.156	-0.954
PB.2312.524(ID1~FSM)	LSC_BC	0.211	-0.988
PB.56688.2(B2M~NNC)	CEC_BC	0.105	-0.989
PB.2386.2(RPN2~NNC)	CC_BC	0.267	-0.967
PB.3524.1636(TUBA1A~FSM)	CEC_CC	0.103	-0.998
PB.13590.41(ENSMFAG00000007224~FSM)	LSC_CC	0.133	-0.996
PB.57004.1(TPM1~NNC)	LSC_CEC	0.369	-0.932
PB.3485.93(SLC38A2~FSM)	CEC_BC	0.198	-0.987
PB.25095.948(ENSMFAG00000006874~NIC)	CEC_CC	0.102	-0.969
PB.16239.382(ZFAND5~NNC)	LSC_BC	0.342	-0.872
PB.46127.2(GLO1~NNC)	LSC_BC	0.293	-0.929
PB.17926.76(HSPA5~FSM)	LSC_BC	0.309	-0.885
PB.25095.948(ENSMFAG00000006874~NIC)	LSC_BC	0.172	-0.941
PB.49383.34(HOPX~NNC)	CEC_CC	0.12	-0.981

Supplementary Table 4. CDD comparison of the major isoform and DEI amino acid sequences.

Query	E-Value	Bitscore	Accession	Short name	Superfamily
Major	6.66E-53	174.497	cl41729	KLF1_2_4_N superfamily	-
Major	7.09E-05	43.531	COG5048	COG5048	cl34881
Diff	6.46E-117	346.682	cd21582	KLF4_N	cl41729
Diff	9.16E-06	42.3584	pfam13465	zf-H2C2_2	cl22375
Diff	4.56E-05	45.8422	COG5048	COG5048	cl34881

Supplementary Table 5. Threshold of "min.pct" and No. of DEIs.

Threshold	0.25	0.2	0.15	0.1
Multi.gene	0	0	2	15
Multi.major	0	0	3	9
Pair.gene	13	24	40	110
Pair.major	3	6	10	35

Supplementary Table 6. CDD comparison of the major isoform and DEI amino acid sequences.

Query	E-Value	Bitscore	Accession	Short name	Superfamily
Major	5.62E-77	246.302	pfam16909	VPS13_C	cl25193
Major	7.21E-15	75.8041	cl05933	SHR-BD superfamily	-
Major	0.00932	35.35	cl47775	WWE superfamily	-
Major	0.009787	39.1639	cl11019	Apt1 superfamily	-
Diff	6.38E-41	135.001	pfam12624	Chorein_N	cl14987
Diff	5.34E-07	48.4095	cl38505	VPS13 superfamily	-

1.Swamynathan SK, Davis J, Piatigorsky J: **Identification of candidate Klf4 target genes reveals the molecular basis of the diverse regulatory roles of Klf4 in the mouse cornea.** *Invest Ophthalmol Vis Sci* 2008, **49:**3360-3370.

2.Li M, Huang H, Wang B, Jiang S, Guo H, Zhu L, Wu S, Liu J, Wang L, Lan X, et al: **Comprehensive 3D epigenomic maps define limbal stem/progenitor cell function and identity.** *Nat Commun* 2022, **13:**1293.

3.Delp EE, Swamynathan S, Kao WW, Swamynathan SK: **Spatiotemporally Regulated Ablation of Klf4 in Adult Mouse Corneal Epithelial Cells Results in Altered Epithelial Cell Identity and Disrupted Homeostasis.** *Invest Ophthalmol Vis Sci* 2015, **56:**3549-3558.

4.Wang Y, Ge H, Chen P, Wang Y: **Wnt/beta-catenin signaling in corneal epithelium development, homeostasis, and pathobiology.** *Exp Eye Res* 2024, **246:**110022.
